# The Evolutionary Patterns of Genome Size in Ensifera (Insecta: Orthoptera)

**DOI:** 10.3389/fgene.2021.693541

**Published:** 2021-06-23

**Authors:** Hao Yuan, Yuan Huang, Ying Mao, Nan Zhang, Yimeng Nie, Xue Zhang, Yafu Zhou, Shaoli Mao

**Affiliations:** ^1^College of Life Sciences, Shaanxi Normal University, Xi'an, China; ^2^Xi'an Botanical Garden of Shaanxi Province/Institute of Botany of Shaanxi Province, Shaanxi Engineering Research Centre for Conservation and Utilization of Botanical Resources, Xi'an, China

**Keywords:** Ensifera, genome size, C-value, phylogeny, flow cytometry

## Abstract

Genomic size variation has long been a focus for biologists. However, due to the lack of genome size data, the mechanisms behind this variation and the biological significance of insect genome size are rarely studied systematically. The detailed taxonomy and phylogeny of the Ensifera, as well as the extensive documentation concerning their morphological, ecological, behavioral, and distributional characteristics, make them a strong model for studying the important scientific problem of genome size variation. However, data on the genome size of Ensifera are rather sparse. In our study, we used flow cytometry to determine the genome size of 32 species of Ensifera, the smallest one being only 1C = 0.952 pg with the largest species up to 1C = 19.135 pg, representing a 20-fold range. This provides a broader blueprint for the genome size variation of Orthoptera than was previously available. We also completed the assembly of nine mitochondrial genomes and combined mitochondrial genome data from public databases to construct phylogenetic trees containing 32 species of Ensifera and three outgroups. Based on these inferred phylogenetic trees, we detected the phylogenetic signal of genome size variation in Ensifera and found that it was strong in both males and females. Phylogenetic comparative analyses revealed that there were no correlations between genome size and body size or flight ability in Tettigoniidae. Reconstruction of ancestral genome size revealed that the genome size of Ensifera evolved in a complex pattern, in which the genome size of the grylloid clade tended to decrease while that of the non-grylloid clade expanded significantly albeit with fluctuations. However, the evolutionary mechanisms underlying variation of genome size in Ensifera are still unknown.

## Introduction

The haploid DNA content per cell, referred to as the genome size or C value, is a basic biological trait of living organisms (Swift, 1950; Greilhuber et al., 2005). It is typically measured in picograms (pg; 1 pg = 10^−12^ g) or megabase pairs (Mbp) where 1 pg = 978 Mbp of DNA. The genome size of different organisms varies dramatically, spanning more than 200,000-fold among eukaryotes (Gregory, 2001) and with at least 7,000-fold variation among animals (Dufresne and Jeffery, 2011). Genome size has a major impact on a range of fitness-related parameters such as growth, metabolism, life history traits, and for many species also body size (Dufresne and Jeffery, 2011; Alfsnes et al., 2017; Yu et al., 2020). Universally, an increase in genome size is concomitant with an increase in cell size, confirmed in almost all biological groups (Mirsky and Ris, 1951; Horner and Macgregor, 1983; Cavalier-Smith, 1985; Gregory, 2000, 2002a, 2005a; Hardie and Hebert, 2003). An increase in cell size leads to a significant increase in the duration of cell division (Bennett, 1977). Since development at the organism level consists of division and growth at the cell level, rate of development is negatively related to genome size. This negative correlation has been demonstrated in some diploid plants and some insects, crustaceans, salamanders, and mammals (Bennett, 1987; White and Mclaren, 2000; Gregory, 2002b, 2005a; Alfsnes et al., 2017). In addition, in many animals, metabolic rate (Kozłowski et al., 2003; Gardner et al., 2020), body size (Glazier, 2021), chromosome number (Ardila-Garcia and Gregory, 2009), and latitude (Carta and Peruzzi, 2016) and altitude (Reeves et al., 1998; Akbudak et al., 2018) also exhibit well-established correlations with genome size. Although these organism level correlations are not universal across all taxonomic groups, all taxonomic groups at least exhibit correlations between genome size, cell size, and cell division rate.

So far, the genome sizes of 6,222 animals have been recorded in the Animal Genome Size Database (http://www.genomesize.com/), represented by 3,793 vertebrates and 2,429 invertebrates (Gregory, 2020). Despite being the most diverse lineage on earth, the genome size of the insects is recorded in only 1,244 species, indicating that the genome size data of the Insecta are relatively limited. Moreover, more than two-thirds of these 1,244 records from the Diptera (386 records), Coleoptera (278 records), and Hymenoptera (240 records). The Orthoptera is the only group of Insecta with a significantly enlarged genome (Alfsnes et al., 2017). The largest known Orthoptera genome is more than 1C = 16 pg, and the genome of most Acrididae is over 1C = 6 pg, far larger than that of mammals (1.42~5.68 pg), birds (1.67~2.25 pg), and most other insects (0.98~8.90 pg) (Gregory, 2020). However, there are only 76 records of the genome size of Orthoptera, covering only 50 species. Among the 50 Orthoptera species, most of the records of genome size are for the Caelifera, with 40 species and 60 records, while only 10 species and 16 records represent the suborder Ensifera. Tettigoniidae, as the most diverse group of Ensifera, has only three records for genome size.

Genome size is one of the most fundamental biological traits of living organisms, not only containing genetic information (genotypic) but also providing an organism's structural components (nucleotypic) (Glazier, 2021; Johnson et al., 2021). At the same time, genome size represents an important basis for comparative research into genome evolution. The lack of genome size data will seriously hinder evolutionary genomics research in the era of genomics. In recent years, studies on the genome size of Orthoptera have been reported for several groups, including our lab, but these studies have focused on the suborder of Caelifera, especially Acrididae (Mao et al., 2020; Shah et al., 2020; Husemann et al., 2021). Ensifera, one of the two monophyletic suborders of Orthoptera, are characterized by long, thread-like antennae, usually longer than the body, and thus are also known as “long-horned grasshoppers,” and include the familiar insects such as crickets, katydids, wetas, and their relatives (Song, 2018). Ensifera is the most diverse group in Orthoptera, but until now there has been no report on Ensiferan genome size and evolution. Characterizing and quantifying genome size variation among Ensifera and whether there is an evolutionary correlation between their genome size and other morphological traits will help us to further understand this group and its significantly enlarged genomes.

With the development of sequencing technology, especially the application of the third-generation single-molecule real-time technology involved in genome assembly, more and more genomes of non-model organisms are being dissected (Ma et al., 2021; Yang et al., 2021). However, for non-model organisms with large and complex genomes, there are still huge difficulties in completing their genome assembly. Despite the current momentum in genomics, large and complex genome sequencing is not available or affordable in most laboratories. Determining the genome size of these non-model organisms with large and complex genomes will not only provide important basic data but also reliable information for the design of subsequent whole-genome sequencing schemes.

As one of the most diverse groups in Orthoptera, Ensifera are well-understood taxonomically and phylogenetically, providing a strong basis for studying the mechanisms of variation and the biological significance of their genome size. In our study, we used flow cytometry to determine the genome size of 32 species of Ensifera. At the same time, we assembled the mitochondrial genomes of nine Ensifera species and combined this with the mitochondrial genomes present in a public database to construct a phylogenetic tree containing 32 Ensifera species and three outgroups. Based on the inferred phylogenetic trees, we detected the phylogenetic signal of genome size variation, compared and analyzed the evolutionary correlation of genome size with body size and flight ability, and constructed the ancestral state of genome size. The results of this study have important theoretical significance for solving the evolutionary patterns of, and influencing factors on, size variation in insect genomes, and they lay a solid foundation for subsequent research into the genome of Ensifera.

## Materials and Methods

### Specimen Collection

A total of 253 adults from 32 Ensifera species were collected from Shaanxi, Guangxi, Inner Mongolia, and Guizhou provinces of China during July to October 2019. The collection information is shown in Supplementary Table 1. All specimens were transported to the laboratory alive and identified based on morphological descriptions. Then, they were frozen rapidly with liquid nitrogen and the heads of the specimens were collected for flow cytometry.

### Flow Cytometry

Genome size was estimated by flow cytometry according to the methods of Mao et al. (2020), Hare and Johnston (2012), and Hanrahan and Johnston (2011). In brief, the full head or half-head of an adult Ensifera specimen (depending on the size for that species) and a suitable internal standard–the head of a male adult *Periplaneta americana* (PAM; 1C = 3.41 pg) or the red blood cells of male *Gallus domesticus* (GRBC; 1C = 1.165 pg) (Mao et al., 2020)–was placed into 1 mL of cold Galbraith buffer. Then, a 2 mL Kontes Dounce tissue grinder (Kontes Glass Co., Vineland, NJ, USA) with type A pestle was used to grind the cells in order to release the nuclei. After grinding, the unwanted cellular debris were filtered with 37 μm nylon mesh, and the nuclei released from the solution were collected into a 1 mL centrifuge tube, which was centrifuged at 1,000 *g* for 5 min, after which the supernatant was discarded. The precipitate was then suspended in 500 μL of phosphate buffer saline (PBS) in the presence of 10 μg/mL Rnase and stained for 30 min in the cold and dark with a final concentration of 50 μg/mL of propidium iodide (PI). Depending on the availability of live samples, 3–16 replicates were measured per species (i.e., biological replicates) with PAM or GRBC as the standard; for each replicate, at least 10,000 nuclei were measured under each 2C peak with the coefficient of variation (CV) of all 2C peaks <5% using a CyAn ADP flow cytometer (Beckman Coulter, Indianapolis, IN, USA) fitted with the laser tuned to 488 nm (Supplementary Table 2; Additional file 2 in Supplementary Material). DNA content was estimated by comparing the ratio of the mean 2C of the sample with the mean 2C of the standard (Lower et al., 2017).

Genome size (bp) was calculated from DNA content (pg) with the following formula (Dolezel et al., 2003): genome size (bp) = (0.978 × 10^9^) × DNA content (pg), which uses the most accurate conversion factor (DoleŽel and Greilhuber, 2010). Mean estimates were calculated for both females and males of a given species derived from the multiple genome size estimates obtained (Table 1). Student's *t*-tests performed with SPSS Statistics v18.0 software (SPSS Inc., Chicago, IL, USA) were used to test for differences of internal standards (PAM vs. GRBC) in six species (*Conocephalus gladiatus, Metrioptera bonneti, Kuwayamaea brachyptera, Deracantha onos, Mecopoda elongata*, and *Teleogryllus emma*) and differences between the sexes (female vs. male) in eight species (*Ruspolia lineosa, C. gladiatus, M. bonneti, Elimaea berezovskii, K. brachyptera, Phaneroptera gracilis, D. onos*, and *M. elongata*).

**Table 1:** Genome sizes (pg) of males and females of 32 Ensifera species, determined using flow cytometry.

**Species**	**Sex**	**N**	**AVG**	**Med**	**SE**	**Min**	**Max**
*Ruspolia lineosa*	Female	6	9.828	9.698	0.135	9.529	10.271
	Male	6	9.089	9.023	0.095	8.809	9.410
*Ruspolia dubia*	Female	8	9.679	9.685	0.082	9.413	10.111
	Male	1	9.066	9.066	NA	9.066	9.066
*Pseudorhynchus crassiceps*	Female	3	10.047	10.049	0.006	10.037	10.055
	Male	3	8.772	8.831	0.102	8.573	8.912
*Conocephalus melaenus*	Female	5	4.301	4.339	0.037	4.207	4.395
	Male	0	NA	NA	NA	NA	NA
*Conocephalus gladiatus*	Female	10	4.541	4.505	0.047	4.401	4.862
	Male	6	4.023	4.009	0.023	3.965	4.130
*Conocephalus maculatus*	Female	2	3.988	3.988	0.095	3.893	4.084
	Male	2	3.687	3.687	0.021	3.666	3.708
*Metrioptera bonneti*	Female	8	5.896	5.857	0.062	5.622	6.148
	Male	8	5.339	5.334	0.045	5.163	5.532
*Tettigonia chinensis*	Female	7	6.680	6.545	0.093	6.442	7.002
	Male	0	NA	NA	NA	NA	NA
*Atlanticus sinensis*	Female	3	7.128	7.115	0.054	7.041	7.227
	Male	5	6.780	6.813	0.067	6.534	6.925
*Gampsocleis sinensis*	Female	4	6.784	6.791	0.043	6.684	6.868
	Male	0	NA	NA	NA	NA	NA
*Elimaea berezovskii*	Female	6	6.680	6.656	0.072	6.454	6.986
	Male	5	5.942	5.777	0.110	5.767	6.311
*Kuwayamaea brachyptera*	Female	7	10.577	10.384	0.172	10.068	11.138
	Male	9	9.055	9.020	0.043	8.883	9.220
*Phaneroptera gracilis*	Female	5	6.113	6.149	0.031	6.016	6.170
	Male	9	5.096	5.114	0.037	4.940	5.281
*Ruidocollaris sinensis*	Female	2	7.002	7.002	0.030	6.971	7.032
	Male	2	6.026	6.026	0.036	5.990	6.062
*Ducetia japonica*	Female	4	7.840	7.789	0.112	7.655	8.129
	Male	4	6.972	6.907	0.106	6.797	7.278
*Zichya tenggerensis*	Female	4	13.952	13.901	0.124	13.723	14.282
	Male	5	12.706	12.776	0.144	12.263	13.068
*Deracantha onos*	Female	6	19.135	19.092	0.157	18.540	19.599
	Male	5	17.393	17.455	0.115	17.084	17.697
*Microconema clavata*	Female	2	4.377	4.377	0.035	4.342	4.412
	Male	2	4.040	4.040	0.027	4.012	4.067
*Tegra novaehollandiae viridinotata*	Female	3	3.473	3.435	0.077	3.363	3.620
	Male	0	NA	NA	NA	NA	NA
*Phyllomimus sinicus*	Female	3	5.913	5.913	0.023	5.873	5.953
	Male	0	NA	NA	NA	NA	NA
*Mecopoda elongata*	Female	6	14.581	14.475	0.248	13.719	15.485
	Male	7	13.453	13.435	0.119	13.037	13.982
*Hexacentrus unicolor*	Female	2	14.008	14.008	0.144	13.864	14.152
	Male	6	12.801	12.753	0.204	12.135	13.666
*Gryllotalpa orientalis*	Female	6	4.205	4.193	0.046	4.055	4.338
	Male	0	NA	NA	NA	NA	NA
*Teleogryllus emma*	Female	9	2.612	2.596	0.023	2.554	2.780
	Male	4	2.341	2.342	0.025	2.283	2.399
*Loxoblemmus equestris*	Female	4	2.446	2.454	0.027	2.376	2.502
	Male	0	NA	NA	NA	NA	NA
*Gryllodes sigillatus*	Female	4	2.271	2.275	0.009	2.248	2.286
	Male	3	2.069	2.082	0.018	2.034	2.091
*Xenogryllus marmoratus*	Female	2	2.351	2.351	0.006	2.344	2.357
	Male	6	2.087	2.096	0.029	1.995	2.157
*Truljalia hibinonis*	Female	0	NA	NA	NA	NA	NA
	Male	4	2.229	2.226	0.032	2.170	2.295
*Oecanthus sinensis*	Female	2	1.081	1.081	0.009	1.072	1.091
	Male	3	0.952	0.969	0.022	0.909	0.979
*Ornebius kanetataki*	Female	3	3.484	3.473	0.011	3.473	3.507
	Male	3	3.082	3.046	0.046	3.027	3.174
*Diestrammena* sp.	Female	2	5.477	5.477	0.003	5.474	5.480
	Male	2	5.145	5.145	0.033	5.112	5.178
*Ocellarnaca* sp.	Female	5	9.451	9.474	0.051	9.306	9.600
	Male	0	NA	NA	NA	NA	NA

*N, Number; AVG, Average value; Med, Median value; SE, Standard error; Min, Minimum value; Max, Maximum value; NA, Missing data*.

### Morphological Measurements

Body size and forewing length were measured from 88 ethanol-preserved adult specimens representing 22 Tettigoniidae species (Supplementary Table 3). As the body size of females varies with the ovulation cycle, we used the length of the hind femur as an indicator of body size (Chapman, 1990; Hochkirch and Gröning, 2008). The femur length is a good proxy for adult body size, confirmed in many studies (Laiolo et al., 2013; Bidau et al., 2016; Anichini et al., 2017; García-Navas et al., 2017). Specimens were photographed on 1-mm grids using a VHX-6000 digital microscope (Keyence, Osaka, Japan) and hind femur length and forewing length were measured. Due to the limited number of specimens, the length of the hind femur and forewing of five species (*Conocephalus melaenus, Atlanticus sinensis, Gampsocleis sinensis, Tegra novaehollandiae viridinotata*, and *Phyllomimus sinicus*) were obtained from the literature (Supplementary Table 3). The mean lengths of the hind femur and forewing in female and male species were shown in Supplementary Table 4.

### DNA Extraction, Sequencing, and Mitogenome Assembly

Genomic DNA of nine Ensifera species (Supplementary Table 5) was isolated from the leg muscle tissue of one individual per species using the TIANamp Genomic DNA Kit (Tiangen Biotech, Beijing, China) following the manufacturer's protocols. DNA libraries were prepared using the NEB Next Ultra DNA Library Prep Kit for Illumina (NEB, Ipswich, MA, USA) according to the manufacturer's instructions, and 150 bp paired-end reads were sequenced on the Illumina Hiseq X 10 platform, obtaining 4 Gb of raw data for each species (Supplementary Table 5). The library preparation and sequencing was completed by the Biomarker Technology Company, Beijing, China.

The mitogenomes were *de novo* assembled based on raw reads using MitoZ v.2.4-alpha software (Meng et al., 2019) with the “all” module. First, raw reads were filtered with a Perl script to obtain clean reads, and clean reads were assembled *de novo* using SOAPdenovo-Trans (Xie et al., 2014) with the quick mode (-K 71). Then HMMER v.3.1b2 (Wheeler and Eddy, 2013) was utilized to construct profile Hidden Markov Models (profile HMMs), and candidate mitogenome sequences were screened based on them. The protein-coding genes (PCGs) were annotated by BLAST v.2.2.19 (Gertz et al., 2006) and GeneWise v.2.2.0 (Birney et al., 2004) using homologous prediction, and tRNA and rRNA were annotated by MiFFi (Jühling et al., 2011) and infernal-1.1.1 (Nawrocki and Eddy, 2013). The mitogenomes of all new sequences have been deposited in GenBank with the accession numbers listed in Supplementary Table 5.

### Phylogenetic Analysis

Phylogenetic trees were constructed using the nucleotide sequences of 13 PCGs and two rRNAs (rrnL and rrnS) from 35 mitogenomes including 32 Ensiferan species and three Caeliferan outgroups (*Atractomorpha sinensis, Tetrix japonica*, and *Locusta migratoria migratoria*) (Supplementary Table 6). All 13 PCGs were codon-based, aligned using ClustalW in MEGA7.0 software (Kumar et al., 2016) and then reverted back to their nucleotide sequences, and the two rRNAs were individually aligned with ClustalW using the default settings. The alignments of the 15 genes were concatenated into a single data matrix using SequenceMatrix v.1.8 (Vaidya et al., 2011). A total of 41 data blocks (13 PCGs divided into individual codon positions and two rRNAs) were partitioned from the single data matrix, and then PartitionFinder v2.1.1 (Lanfear et al., 2012) using the “greedy” algorithm (heuristic search) and “unlinked” branch lengths was used to identify the best-fit partitioning scheme for the data blocks, and to estimate the optimal nucleotide substitution model for each partition.

We used maximum likelihood (ML) and Bayesian inference (BI) methods to perform phylogenetic inference and assess the support of the clades. ML analysis was performed by RaxML v8.2.12 (Stamatakis, 2014) with the optimal partitions and best models selected by PartitionFinder2 (Lanfear et al., 2012), and 1,000 replicates to evaluate node support. BI analysis was performed by MrBayes v3.2 (Ronquist et al., 2012), and also by employing the optimal partitions and best models selected by PartitionFinder2, with four Markov Chain Monte Carlo (MCMC) chains for 10,000,000 generations. Each set was sampled every 1,000 generations, the first 25% of generations were discarded as burn-in, and the remaining samples were used to obtain the consensus tree. We also performed a divergence time estimate analysis using BEAST v1.10.4 (Suchard et al., 2018) to estimate the relative time of divergence of the studied taxa. The optimal partitions and nucleotide evolution models were recommended by PartitionFinder2. For each partition an uncorrelated lognormal relaxed-clock was implemented, and the tree prior was set to a Yule process. Three calibration points were used to impose age constraints of the tree. The first calibration point we selected was the fossil of *Raphogla rubra* Béthoux, 2002, known from the Permian of France [260.4–251 million years ago (Mya)] (Bethoux et al., 2002). This is the oldest definitive fossil of Ensifera as recognized by several previous studies (Song et al., 2015; Zhou et al., 2017; Li et al., 2019). The second calibration point we selected was the fossil of *Gryllus vociferans* Cockerell (1925), known from the Margas Verdes Formation of the Paleocene of Argentina (66.0–56.0 Mya) (Cockerell, 1925). *G. vociferans* is the oldest definitive fossil of Gryllinae, which we used to calibrate the clade of Gryllinae. The final calibration point we selected was the fossil of *Conocephalus martyi* (Piton, 1940), known from the Menat Formation of the Paleocene of France (59.2–56.0 Mya) (Piton, 1940), which is the oldest known fossil of Conocephalinae (Zhou et al., 2017; Li et al., 2019). For the BEAST analysis, we ran 1 × 10^9^ generations with sampling every 1,000 generations, and a burn-in of 25% of the trees. Tracer v.1.6 (Rambaut and Drummond, 2009) was used to inspect the results and confirmed that all parameters were achieved ESS > 200. TreeAnnotator v1.10.4 (Rambaut and Drummond, 2002) was used to summarize the maximum clade credibility (MCC) tree, median ages, and 95% highest posterior density (HPD).

### Phylogenetic Comparative Analyses

To test for phylogenetic signal in the genome size of the 32 Ensifera species, three common metrics, including Pagel's lambda (λ) (Pagel, 1999), Blomberg's *K* (Blomberg et al., 2003), and Abouheif's C_mean_ (Abouheif, 1999) were estimated based on the phylogeny we constructed. Pagel's λ is a branch-length transformation method, estimated by ML to test the best fit of a trait against a Brownian model. The values of Pagel's λ vary from 0 to 1, with 0 indicating no phylogenetic signal and a random distribution of traits, 1 indicating a strong phylogenetic signal and the distribution of traits following the Brownian motion model. λ > 1 is possible because λ is not a correlation but a scaling factor for a correlation. The metrics of Blomberg's *K* are quite different from Pagel's λ. Blomberg's *K* is a variance ratio (a scaled ratio of the variance among species over the contrasts variance), which is rescaled by dividing by the Brownian motion expectation. When *K* < 1, the evolution of traits is independent of the phylogeny, and when *K* > 1 they are dependent of the phylogeny. Abouheif's C_mean_ is another way of testing for phylogenetic signal, however it does not rely on an evolutionary model but rather measures autocorrelation among tips by a specific phylogenetic approximation matrix and can be tested by random permutation. Pagel's λ and Blomberg's *K* were calculated using the phylosig function in the phytools package (Revell, 2012) in R v3.6.1 (Team, 2013), and Abouheif's C_mean_ was calculated using the adephylo package (Jombart and Dray, 2010).

To identify where in the phylogeny the genome size changed, we reconstructed the ancestral state of genome size using three methods: maximum parsimony (MP) analysis using Mesquite v3.61 (Maddison and Maddison, 2015) with default settings, ML analysis using the fastAnc function from the phytools package (Revell, 2012) in R v3.6.1 (Team, 2013), and BI analysis using the MCMC model A (continuous: random walk) in BayesTraits v3.0.2 (Pagel et al., 2004). For BI analysis, we first considered whether genome size evolution followed models A (random walk) or B (directional) by model testing implemented in BayesTraits. The log marginal likelihoods for models A and B were estimated using stepping-stone sampling, running 1,000 stones with 100,000 iterations for each stone, and then a log Bayes factor (BF) was calculated. The model tests of BF indicated that model A was significantly better than model B (BF_female_ = 10.95; BF_male_ = 11.17), so we used model A for the downstream analysis. To estimate the ancestral genome sizes in BayesTraits, the Markov chain model was generated to run 100 million generations, with a burn-in period of 5 million generations and chain sampling every 10,000 generations.

We also examined the phylogenetic relationships among genome size, body size, and forewing length of 22 Tettigoniidae species using phylogenetic generalized least squares (PGLS). Prior to PGLS analysis, we performed an ordinary least squares (OLS) regression analysis (equivalent of PGLS with λ = 0), which assumed phylogenetic independence among all of the traits, using the function lm in the package stats in R v3.6.1. We then performed a PGLS analysis in the R package caper (Orme et al., 2013), using the ML estimation of λ to transform branch lengths. In the PGLS analysis, we used genome size as the response variable, and body size and forewing length as the predictor variables. The time-calibrated tree obtained by BEAST was pruned to the 22 Tettigoniidae for PGLS analysis. All trait values were log transformed before performing PGLS analysis. We also investigated the phylogenetic signal (Pagel's λ, Blomberg's *K*, and Abouheif's C_mean_) of genome size, body size and forewing length measured in the 22 Tettigoniidae species to evaluate the appropriateness of the test for statistical significance.

## Results

### Assembled Mitogenomes of Nine Ensifera Species

We assembled the complete mitogenomes of nine Ensifera species using the Illumina data sequenced in this study (Supplementary Table 5). The size of these mitogenomes ranged from 15,297 bp (*Diestrammena* sp.) to 16,416 bp (*Ruidocollaris sinensis*). All mitogenomes have the typical gene content found in metazoan mitogenomes: 13 PCGs, 22 tRNA genes, two rRNA genes, and one non-coding region (the A+T-rich region). Nine PCGs, 14 tRNAs, and the A+T-rich region are located in the major strand (J-strand), and four PCGs, eight tRNAs, and two rRNAs are located in the minor strand (N-strand). The gene order and orientation of the mitogenome of all nine species are identical to that of the ancestral mt gene. The base composition, A+T content, G+C content, AT skew, and GC skew exhibit similar characteristics in the nine species (Supplementary Table 5).

### Genome Size Estimation of Ensifera

In this study, the genome sizes of 32 Ensifera species were measured by flow cytometry. To accurately estimate the genome size of each species, 3–16 biological replicates were measured per species depending on the availability of living samples, and two internal standards, PAM (1C = 3.41 pg) and GRBC (1C = 1.165 pg), were used for all species except for *Pseudorhynchus crassiceps, C. maculatus, R. sinensis, Microconema clavata, T. novaehollandiae viridinotata, P. sinicus, Loxoblemmus equestris, Gryllodes sigillatus, Truljalia hibinonis, Oecanthus sinensis, Diestrammena* sp., and *Ornebius kanetataki* (Supplementary Table 2; Additional file 2 in Supplementary Material). In addition, we also selected six species to compare the genome size estimated by the two internal standards (PAM vs. GRBC), to test the effect of internal standard on estimated genome size (Supplementary Table 7). The results showed that except for *C. gladiatus* (*P* = 0.011) and *D. onos* (*P* = 0.042) in females and *M. bonneti* (*P* = 0.043) in males, there was no significant difference (*P* > 0.05) in genome size estimated by the two different internal standards of PAM and GRBC. Since multiple genome size estimates were obtained for both females and males of each species, the estimate mean was calculated (Table 1).

Across the estimated genome sizes of the 32 Ensifera species, C-values ranged from 1C = 0.952 pg (the male of *O. sinensis*) to 1C = 19.135 pg (the female of *D. onos*) representing a >20-fold range. Within families, the genomes of Tettigoniidae were generally large, the variation of genome size was above 5.5-fold (3.473–19.135 pg), and the average genome size reached 8.276 pg. The genomes of Gryllidae were relatively small, ranging in size from 1C = 0.952 pg to 1C = 2.612 pg (2.7-fold) with an average genome size of 2.044 pg. For the remaining four families, including Gryllotalpidae, Mogoplistidae, Rhaphidophoridae, and Gryllacrididae, only one species of each family had its genome size measured of which the genome size of females of the *Ocellarnaca* sp. of Gryllacrididae was found to be relatively large, reaching 1C = 9.451 pg.

**Figure 1:**
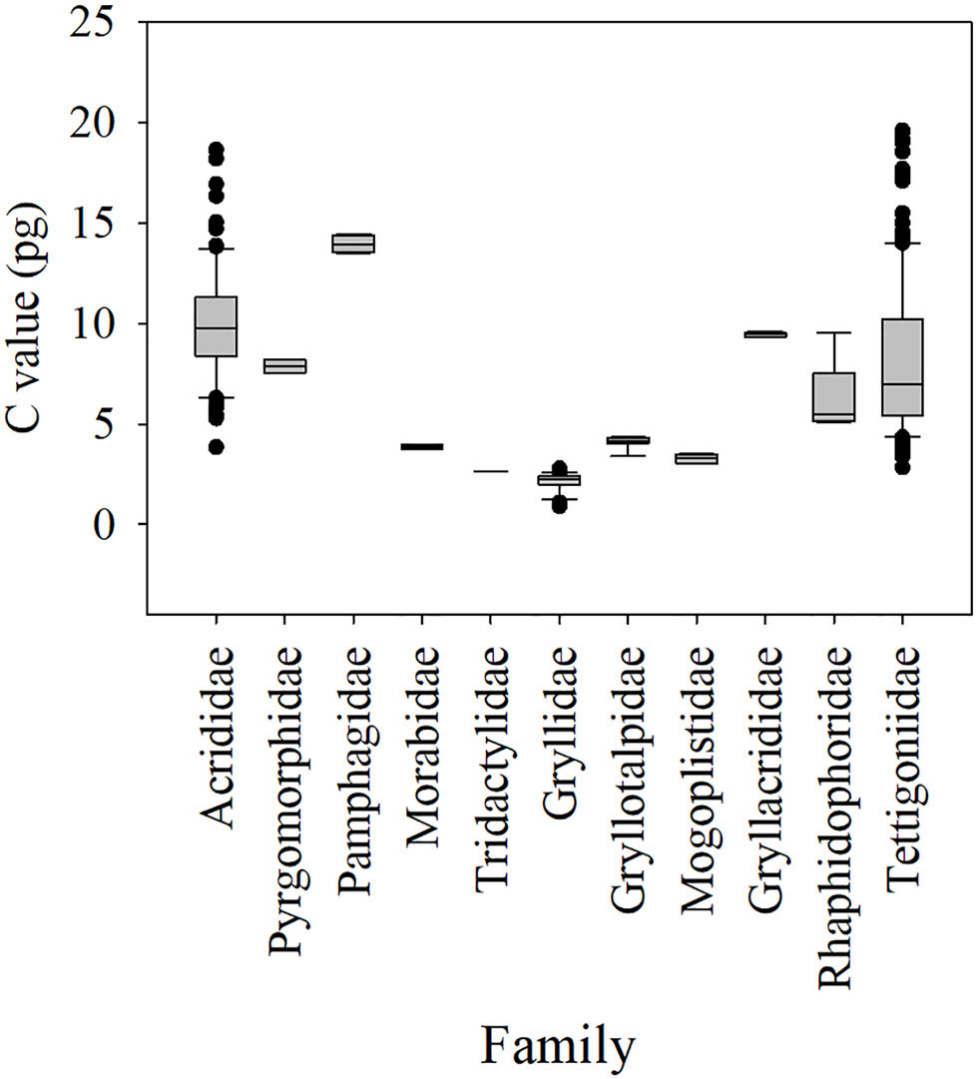
Boxplot of genome sizes for families of Orthoptera, including data from the present study and the Animal Genome Size Database (Gregory, 2020).

At present, there are a total of 76 records (representing 50 species) of the genome size of Orthoptera insects in the Animal Genome Size Database (http://www.genomesize.com), most of which are Acrididae (58 records). The Orthoptera genome size recorded in the database ranged from 1.55 pg in the cave cricket *Hadenoecus subterraneus* to 16.93 pg in the mountain grasshopper *Podisma pedestris*, representing a nearly 11-fold range. All of these are much larger than the smallest genome we have newly measured (the male of *O. sinensis*: 1C = 0.952 pg), and much smaller than the largest genome we have newly measured (the female of *D. onos*: 1C = 19.135 pg). Our study provides a broader blueprint for genome size variation of Orthoptera. To better understand the genome size variation of the Orthoptera in families, we collected the genome size data of Orthoptera from the Animal Genome Size Database and the genome size data of Orthoptera published by Mao et al. (2020), Shah et al. (2020), and Husemann et al. (2021) in recent years. We combined these with the genome size data of 32 newly measured Ensifera species for statistical analysis (Figure 1). The genome sizes of 11 families of Orthoptera were obtained; the genome size variations within the Tettigoniidae and Acrididae each represent a large range. Interestingly, Tettigoniidae and Acrididae are the two most diverse families of Orthoptera and appear to have undergone explosive, adaptive radiation (Song et al., 2015).

### Sex Differences in Genome Size

**Figure 2:**
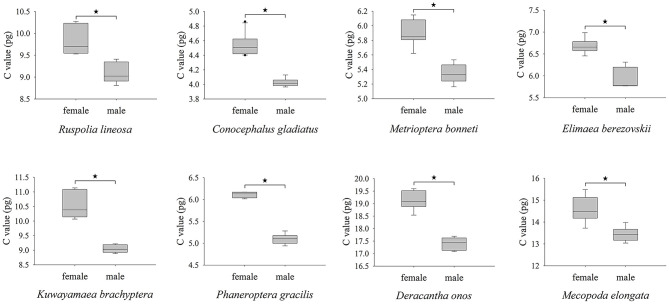
Comparative analysis of the genome sizes of females and males in eight Ensifera species. *Indicates a significant difference between both sexes (*P* < 0.01).

Typically, sex has a significant effect on genome size variation at the within-species level, and the significant effects caused by sex cannot be determined with estimates that ignore or merge values (Hanrahan and Johnston, 2011). In our study, we separated genome size estimates by sex for all species except for a few where sample size was very limited (Table 1; Supplementary Table 2). By analyzing the sex differences of eight species for which we had at least five replicates of each sex (Figure 2; Supplementary Table 8), we found that females had significantly larger genomes than males in every case (*P* < 0.01). In Caelifera, the genome sizes of females are also significantly larger than those of males (Mao et al., 2020). The sex difference in genome size may be caused by the sex chromosome because most Ensifera species are XO sex determined (Warchalowska-Sliwa, 1998). Among the eight species, the genome of females is ~10% larger than that of males, indicating that the X chromosome is ~10% of the genome. Based on this inference, we estimated genome sizes where they were missing: female *T. hibinonis* (1C = 2.452 pg) and male *C. melaenus* (1C = 3.871 pg), *Tettigonia chinensis* (1C = 6.012 pg), *G. sinensis* (1C = 6.106 pg), *T. novaehollandiae viridinotata* (1C = 3.126 pg), *P. sinicus* (1C = 5.322 pg), *Gryllotalpa orientalis* (1C = 3.785 pg), *L. equestris* (1C = 2.201 pg), and *Ocellarnaca* sp. (1C = 8.506 pg). These estimates were used for subsequent analysis.

### Evolutionary Pattern of Genome Size

To explore the evolutionary pattern of genome size within Ensifera, we constructed three phylogenetic trees for the measured species using the mitogenome sequences of 13 PCGs and two rRNAs based on the software packages RaxML, MrBayes, and BEAST. Phylogenies obtained by these packages were consistent and robust, and largely congruent with those reconstructed previously (Zhou et al., 2017; Li et al., 2019) (Supplementary Figures 1–3). The phylogenetic trees were divided into two clades: grylloid (the infraorder Gryllidea) and non-grylloid. In the grylloid clade there were eight species, six of which were Gryllidae, categorized into a monophyletic group. In the non-grylloid species, Tettigoniidae was the most diverse group with 22 species, also belonging to a monophyletic group. The divergence time analysis showed that the split between grylloid and non-grylloid occurred at the Permian/Triassic boundary, and both Gryllidae and Tettigoniidae originated in the Late Jurassic and diversified into major lineages in the Cretaceous. This estimated timescale is consistent with previous studies (Song et al., 2015; Li et al., 2019).

Based on the inferred phylogenetic relationship, we estimated Pagel's λ, Blomberg's *K*, and Abouheif's C_mean_ to determine the phylogenetic signal of genome size variation. In the analysis of 32 Ensifera species, both male and female genome sizes exhibited strong phylogenetic signals (female: Pagel's λ = 1.028, Blomberg's *K* = 0.769, Abouheif's C_mean_ = 0.444; male: Pagel's λ = 1.045, Blomberg's *K* = 0.770, Abouheif's C_mean_ = 0.440), indicating that the genome size of related species was more similar than expected under Brownian motion. In the analysis of 22 species of Tettigoniidae, there were also strong phylogenetic signals for the genome size of males and females (female: Pagel's λ = 1, Blomberg's *K* = 0.840, Abouheif's C_mean_ = 0.221; male: Pagel's λ = 1, Blomberg's *K* = 0.854, Abouheif's C_mean_ = 0.222), but the phylogenetic signals were significantly smaller than those of the 32 Ensifera species in terms of Abouheif's C_mean_, which might be influenced by the number of taxa sampled (see Supplementary Table 9 for details).

We also assessed the evolution of body size and forewing length of the 22 Tettigoniidae species using phylogenetic signals. We found that both males and females had strong phylogenetic signals in their forewing length (female: Pagel's λ = 1, Blomberg's *K* = 0.799, Abouheif's C_mean_ = 0.266; male: Pagel's λ = 1, Blomberg's *K* = 0.801, Abouheif's C_mean_ = 0.292), whereas body size showed only moderate phylogenetic signals (female: Pagel's λ = 1, Blomberg's *K* = 0.698, Abouheif's C_mean_ = 0.149; male: Pagel's λ = 0.78, Blomberg's *K* = 0.685, Abouheif's C_mean_ = 0.139) (Supplementary Table 9). With the 22 Tettigoniidae species, we tested whether the differences in inter-species genome size were related to flight ability in terms of forewing length (where a longer forewing is generally considered to be associated with stronger flight ability) and body size, using OLS and PGLS regression analysis. We found no correlation between genome size and flight ability or body size in both OLS and PGLS regression analyses (Supplementary Table 10).

**Figure 3:**
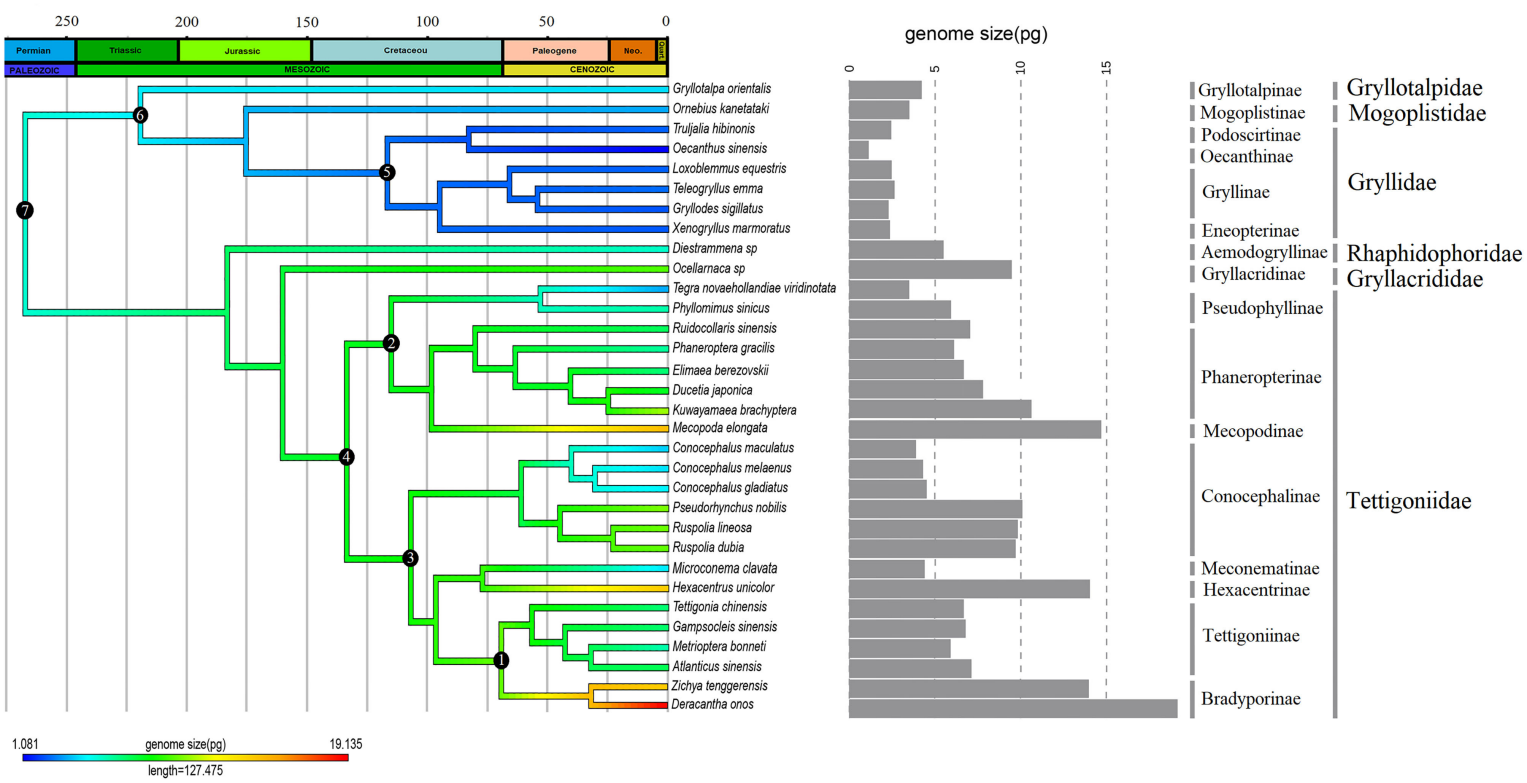
Ancestral reconstruction of the genome size of (female) Ensifera based on a time-calibrated phylogenetic tree. The black circles with numbers on the nodes correspond to the estimated ancestral genome size using fastAnc, BayesTrait, and Mesquite in Table 2. The average genome size of each species is shown in a histogram on the right.

The genome sizes of the ancestral nodes of Ensifera species were estimated based on the inferred phylogenetic relationship using three methods: MP, ML, and BI (Figure 3; Table 2; Supplementary Figure 4). For females, the genome size of the most common ancestor of Ensifera (Node-07) reconstructed using MP was 5.909 pg, using ML was 5.354 pg, and using BI was 5.372 ± 0.938 pg. For males, the ancestral genome size of Ensifera (Node-07) reconstructed using MP, ML, and BI was 5.367, 4.843, and 4.875 ± 0.910 pg, respectively. In comparing the three methods of ancestral state reconstruction, the results of the MP method were found to be slightly different from those of the ML method and BI method, while there was no difference between the ML and BI methods, and the results of the ML and BI methods were more reliable (Table 2). Across the ancestral state reconstruction of Ensifera genome size, we found several increasing and decreasing events. In the ancestral lineages of the grylloid clade, the genome sizes underwent a dramatic decrease, while in the ancestral lineages of the non-grylloid clade, the genome sizes increased (Figure 3; Table 2; Supplementary Figure 4). For the grylloid clade, the genome size of the ancestral node (Node-06) was 4.469 ± 1.224 pg (female, BI method) and then decreased to 2.693 ± 0.980 pg (the ancestral node of Gryllidae [Node-05, female, BI method]), while the genome size of *O. sinensis* was the smallest, decreasing to 1.081 pg (female). For the non-grylloid clade, there was a significant expansion in genome size. Up to the ancestral node of Tettigoniidae (Node-04), genome size had increased to 7.975 ± 0.852 pg (female, BI method), and *D. onos* had the largest expansion, reaching 19.135 pg (female). In addition to the expansion of genome size, there were multiple evolutionary transitions from a very large ancestral genome size to a smaller genome size in the non-grylloid clade. For example, there were three independent lineages in the Tettigoniidae that transitioned to a smaller genome size—*Tegra, Conocephalus*, and *Microconema*—all of which have genome sizes smaller than 5 pg. However, genome size expansion from the ancestral state is a general trend in the non-grylloid clade.

**Table 2:** The estimated genome size in seven ancestral nodes inferred using fastAnc, BayesTrait, and Mesquite.

	**Node**	**fastAnc (pg)**	**BayesTraits (pg)**	**Mesquite (pg)**
Female	Node-01	9.725	9.721 ± 0.731	9.618
	Node-02	7.931	7.950 ± 0.842	8.120
	Node-03	8.441	8.461 ± 0.787	8.114
	Node-04	7.974	7.975 ± 0.852	7.910
	Node-05	2.674	2.693 ± 0.980	3.046
	Node-06	4.447	4.469 ± 1.224	5.309
	Node-07	5.354	5.372 ± 0.938	5.909
Male	Node-01	8.851	8.837 ± 0.666	8.751
	Node-02	7.136	7.133 ± 0.768	7.320
	Node-03	7.661	7.664 ± 0.712	7.364
	Node-04	7.205	7.205 ± 0.785	7.160
	Node-05	2.402	2.407 ± 0.864	2.749
	Node-06	4.011	4.054 ± 1.082	4.814
	Node-07	4.843	4.875 ± 0.910	5.367

*All values of genome size in BayesTrait analysis show the average ± one standard deviation (ave ± sd)*.

*#Node-01, (Tettigoniinae+Bradyporinae); #Node-02, ((Phaneropterinae+ Mecopodinae)+Pseudophyllinae); #Node-03, (((Tettigoniinae+Bradyporinae)+ (Hexacentrinae+Meconematinae)) +Conocephalinae); #Node-04, ((((Tettigoniinae+ Bradyporinae) +(Meconematinae+Hexacentrinae)) +Conocephalinae) +((Phaneropterinae+ Mecopodinae)+Pseudophyllinae)); #Node-05, ((Gryllinae+Eneopterinae) + (Oecanthinae+Podoscirtinae)); #Node-06, ((((Gryllinae+ Eneopterinae) + (Oecanthinae+Podoscirtinae))+Mogoplistinae)+Gryllotalpinae); #Node-07, root*.

## Discussion

Although our knowledge of genome sizes in insects is steadily increasing, our knowledge of the suborder Ensifera lags behind. Prior to our study, there were only 10 species and 16 records of Ensifera in the Animal Genome Size Database (http://www.genomesize.com) and most of these were Gryllidae (five species and 11 records). These published data provided only limited information on the pattern of genome size diversity in Ensifera. In our study, we measured the genome size of 32 species of Ensifera using flow cytometry (Table 1; Supplementary Table 2; Additional file 2 in Supplementary Material), which served to considerably increase the number of known genome sizes of Ensifera and provided data for studying the evolution of this suborder's genome size; the first study of its kind for Ensifera. Compared with all other insects in the Animal Genome Size Database, the genome size of Ensifera is very large with considerable variation. The smallest genome size was found in Gryllidae (the male of *O. sinensis*: 1C = 0.952 pg), and the largest in Tettigoniidae (the female of *D. onos*: 1C = 19.135 pg). The difference between the largest and smallest genome size was over 20-fold. At the same time, the results of ancestral state reconstruction inferred using Mesquite, fastAnc and BayesTraits revealed that the genome size of Ensifera has evolved in complex ways (Figure 3; Table 2; Supplementary Figure 4). The genome size of the grylloid clade decreased while the genome size of the non-grylloid clade increased significantly, with fluctuations.

Genome downsizing may be due to unequal intra-strand homologous recombination, double-strand break repair, illegitimate recombination or retroelement extinction (Chen et al., 1998; Vicient et al., 1999; Shirasu et al., 2000; Orel and Puchta, 2003; Dufresne and Jeffery, 2011). The removal of extra DNA is thought to avoid the extra metabolic costs needed to maintain the harmonization of genome constituents, an energy efficiency that should favor species survival (Gregory and Hebert, 1999). Gryllidae form the second most diverse clade of the ancient lineages within Ensifera, with more than 4,800 known species (Eades, 2000). Recent studies have found no major shifts in the rate of diversification in Gryllidae, which have continually diversified since the origin of the clade (Song et al., 2015). This consistency of diversification rate may be related to the continuous decrease of the Gryllidae genome size. Studies have shown that genome size reductions can sometimes be followed by low rates of extinction (Volff, 2005; Organ et al., 2007; Gregory et al., 2009).

Tettigoniidae represent the most successful lineage within Ensifera in terms of species diversity with over 7,500 known species and may have undergone explosive adaptive radiation (Mugleston et al., 2018). Our study found that the genome size of Tettigoniidae is extremely large, with the average genome size reaching 8.276 pg and the largest exceeding 19 pg—the largest known genome in the Insecta. Genome expansion events usually include small-scale insertions of nucleotides or large-scale alterations such as gene duplications, transposon insertions, or polyploidy (Petrov, 2001; Dufresne and Jeffery, 2011). Although these are very different processes, they have the potential to influence rates of diversification and promote the formation of new species (Kraaijeveld, 2010). Large-scale duplication events are at the base of a number of evolutionary radiations, such as the radiation of teleost fish (Volff, 2005). The radiation evolution of the family Tettigoniidae may be related to the expansion of its genome size, and a large-scale duplication event may have occurred in the early stages of Tettigoniidae radiation.

In our study, we used three methods (Pagel's λ, Blomberg's *K*, and Abouheif's C_mean_) to determine the phylogenetic signals in the genome size variation of 32 Ensifera (Supplementary Table 9). We determined that there was a strongly phylogenetic signal in the genome size of Ensifera species, indicating a tendency for it to be more similar among related species than genome size would be within a random set of species from the same tree (Harvey and Pagel, 1991). Similarly strong phylogenetic signals for genome size have been detected in other insects such as grasshoppers and locusts (Orthoptera, Acrididae) (Mao et al., 2020), fireflies (Coleoptera, Lampyridae) (Lower et al., 2017), butterflies (Lepidotera, Papilionoidea) (Liu et al., 2020), and fruit flies (Diptera, Drosophilidae) (Hjelmen and Johnston, 2017). These findings suggest that genome size is phylogenetically conserved among closely related species, which may be the result of similar gene content and stretches of conserved gene order (Kellogg and Bennetzen, 2004). Meanwhile, we also determined phylogenetic signals for genome size variation in 22 Tettigoniidae (Supplementary Table 9). We found that the phylogenetic signals determined by 32 Ensifera were different from those for 22 Tettigoniidae, especially the value of Abouheif's C_mean_, which might be influenced by the number of taxa sampled. Current studies have shown that the method of Abouheif's C_mean_ is sensitive to taxa number and the measured phylogenetic signal increases with a significant increase in taxa number, whereas Pagel's λ and Blomberg's *K* are less sensitive to taxa number (Hjelmen and Johnston, 2017). It is therefore suggested that the Abouheif's C_mean_ can only be used as a preliminary test for the determination of phylogenetic signals while Pagel's λ and Blomberg's *K* can be considered as definitive measures. In addition, sample sizes of at least 15 are necessary to achieve reliable results in terms of significance for Pagel's λ and Blomberg's K (Hjelmen and Johnston, 2017); our sample size is substantially larger than this.

Morphological measurements of body size and forewing length were also undertaken for 22 Tettigoniidae species (Supplementary Tables 3, 4). We expected to find a positive correlation between genome size and body size, which is assumed to be caused by nucleotypic effects of cell size/volume (Cavalier-Smith, 1978; Gregory, 2005b). However, our PGLS analysis did not find evidence of a positive correlation between genome size and body size (Supplementary Table 10). This is not unusual, for example previous studies of ladybird beetles (Coleoptera, Coccinellidae) (Ryan Gregory et al., 2003) and fireflies (Coleoptera, Lampyridae) (Lower et al., 2017) in Insecta have also not found correlations. Clearly, then, there is not necessarily evolutionary correlation between genome size and body size.

In vertebrates, the association between genome size and flight ability has become increasingly apparent, and strong fliers exhibit smaller genomes than do weak fliers or flightless birds (Hughes, 1999; Gregory, 2005b). In insects, although this relationship has not been well-studied, genome size is related to the flight strategy of dragonflies, with the average genome of “fliers” being much larger than that of “perchers” (Ardila-Garcia and Gregory, 2009). However, the category “fliers” and “perchers” is not a taxonomic artifact of including different suborders in the comparison. In our study, the PGLS analysis found no evidence of a correlation between genome size and flight ability (forewing length) (Supplementary Table 10). Current studies of fireflies have also found that these two variables do not correlate (Lower et al., 2017). However, our sample size is small and more species are needed to rigorously test the correlation between genome size and flight ability, including body size.

In recent decades, it has been recognized that the main mechanism contributing to genome size variation is repetitive DNA, including whole genome duplication (WGD), chromosome aneuploidy/supernumerary, indels, gene duplications/deletions, and transposable elements (TEs) (Petrov, 2001; Gregory, 2005b,c). Polyploidy or WGD is a major contributor to genome evolution and diversity (Li et al., 2018). A recent study reported that ancient polyploidy was found in the ancestors of some insects, including Lepidoptera, Trichoptera, and Odonata, but not in Orthoptera (Li et al., 2018). This indicates that the very large genome of Orthoptera is not caused by paleopolyploidy. Although there has been no report so far on the repetitive DNA content of Ensifera, studies of the *L. migratoria* genome (Wang et al., 2014), the *Schistocerca gregaria* genome (Verlinden et al., 2020), and the repetitive elements of several other orthopteran insects (Camacho et al., 2015; Ruiz-Ruano et al., 2018; Shah et al., 2020) have revealed that the proportion of repeats in the large genome of orthopteran insects is very high. In *L. migratoria*, repetitive elements constituted ~60% of the assembled genome, and the very large genome of *L. migratoria* may be caused by the continuous proliferation of repetitive elements combined with slow rates of loss for these elements (Wang et al., 2014). The genome size of Ensifera, especially Tettigoniidae, has significantly expanded, which may also be the result of the continuous proliferation of repetitive elements in the genome.

The expansion of the genome is not out of control. Studies have uncovered the Piwi-interacting RNA (piRNA) pathway, a small RNA genome defense system that suppresses TE activity in the animal germline (Mueller, 2017). piRNAs limit the expansion of genome size by guiding the transcription and post-transcriptional silencing of TEs through base complementarity. In addition to piRNA, studies have shown that mate choice can also regulate genome size. In the study of the bow-winged grasshopper *Chorthippus biguttulus*, males with smaller genomes had more attractive songs and females were more likely to mate with them (Schielzeth et al., 2014). Through this courtship mechanism, the continuous enlargement of the genome is to some extent controlled. The results of ancestral state reconstruction showed that the genomes of the grylloid clade in Ensifera shrank significantly, especially in the Gryllidae. What causes the shrinking of the grylloid clade genome? This is a question worthy of in-depth study, which our future research will focus on.

In conclusion, our study is the first extensive survey of genome size variation within the suborder of Ensifera. We measured the genome size of 32 species of Ensifera using flow cytometry and found that it has an overall 20-fold range from 0.952 to 19.135 pg; our data provide a broader blueprint for genome size variation in Orthoptera. However, the species of Ensifera for which we have genome size data only account for a small percentage of all of the suborder's species. Thus, more species still need to be investigated in order to systematically and comprehensively explore the evolution of Ensifera genome sizes. Nonetheless, the genome size data obtained in this study and the corresponding findings will still be very helpful for in-depth study of the mechanisms of genome size increase and decrease within the Ensifera.

## Data Availability Statement

The datasets presented in this study can be found in online repositories. The names of the repository/repositories and accession number(s) can be found at: NCBI [accession: MT849265-MT849273].

## Author Contributions

HY: conceptualization, methodology, software, formal analysis, investigation, data curation, writing–original draft, writing–review and editing, and visualization. YH: conceptualization, resources, writing–review and editing, project administration, funding acquisition, and supervision. YM: conceptualization, methodology, software, and investigation. NZ: methodology, software, and formal analysis. YN: data curation, methodology, and formal analysis. XZ: data curation and methodology. YZ: investigation, writing-review and editing. SM: conceptualization, methodology, investigation, resources, writing-review and editing, project administration, funding acquisition, and supervision. All authors contributed to the article and approved the submitted version.

## Conflict of Interest

The authors declare that the research was conducted in the absence of any commercial or financial relationships that could be construed as a potential conflict of interest.
