# Supplementary material for: The Evolutionary Patterns of Genome Size in Ensifera (Insecta: Orthoptera)
Source: Front Genet. 2021 Jun 23;12:693541. doi: 10.3389/fgene.2021.693541 (PMC8261143; doi:10.3389/fgene.2021.693541)

Figure S1 Phylogeny of Ensifera inferred from maximum likelihood analysis using the mitogenome sequences of 13 PCGs and two rRNAs (rrnL and rrnS). Values indicated on nodes represent bootstrap support (1,000 replications).

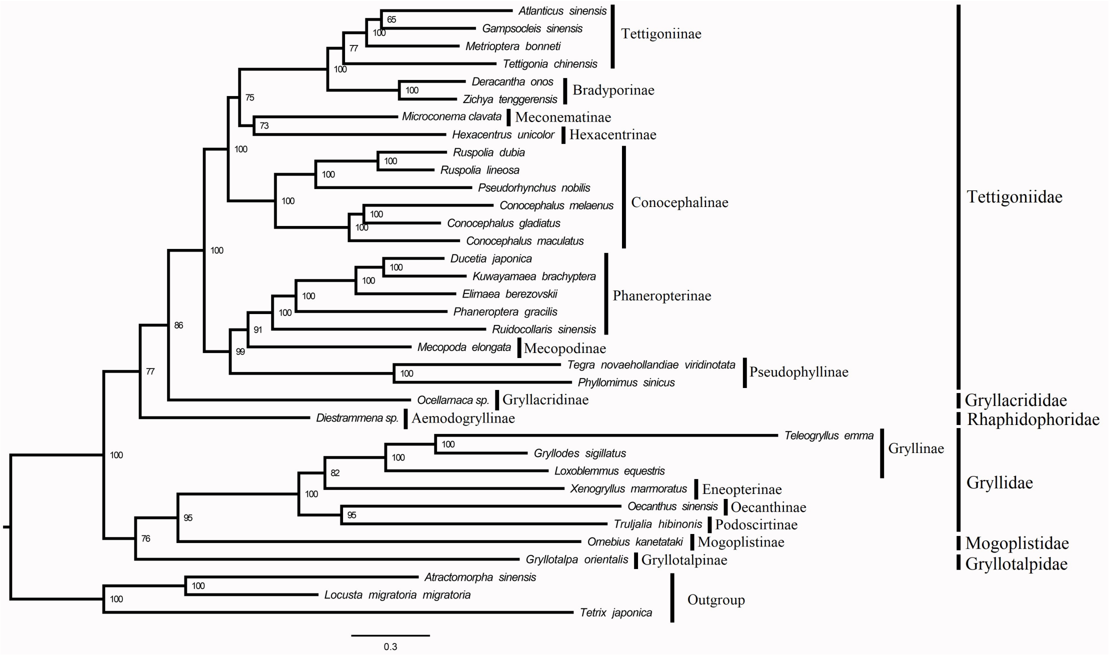

Figure S2 Phylogeny of Ensifera based on Bayesian inference analysis using the mitogenome sequences of 13 PCGs and two rRNAs (rrnL and rrnS). Values indicated on nodes represent Bayesian posterior probabilities (10,000,000 generations; sample frequency = 1,000 generations; burn-in = 25%).

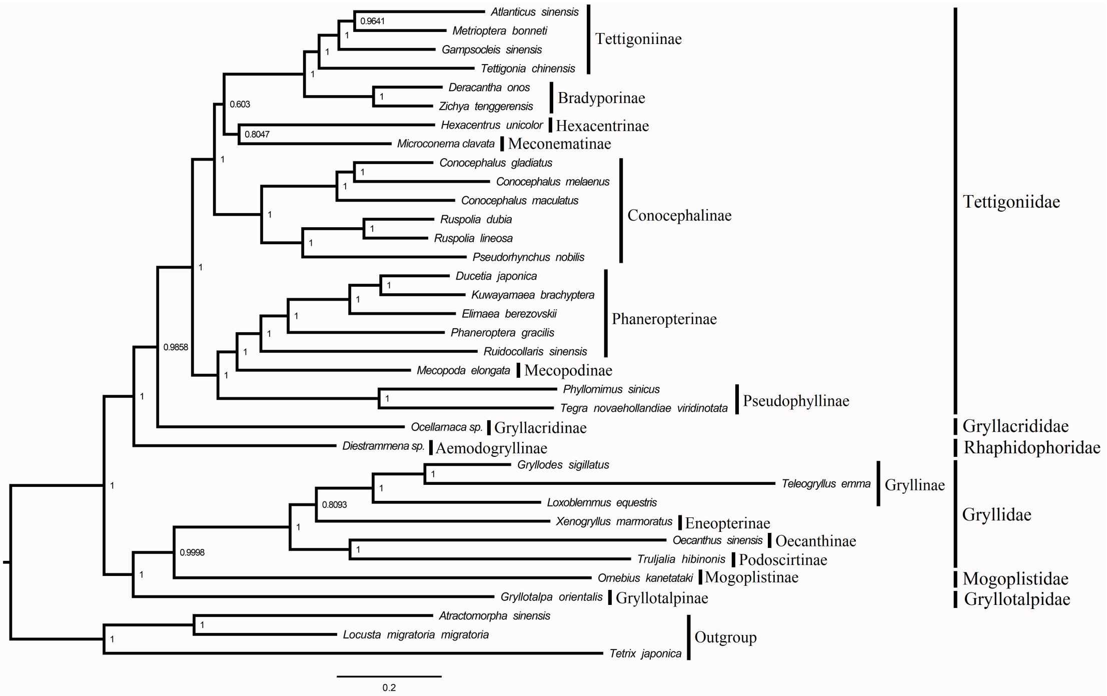

Figure S3 Time-divergence estimates of Ensifera lineages using three fossil calibration points. Blue bars indicate 95% median confidence intervals. White circles on nodes indicate posterior probability values less than 95. Yellow circles on nodes denote the fossil calibration points.

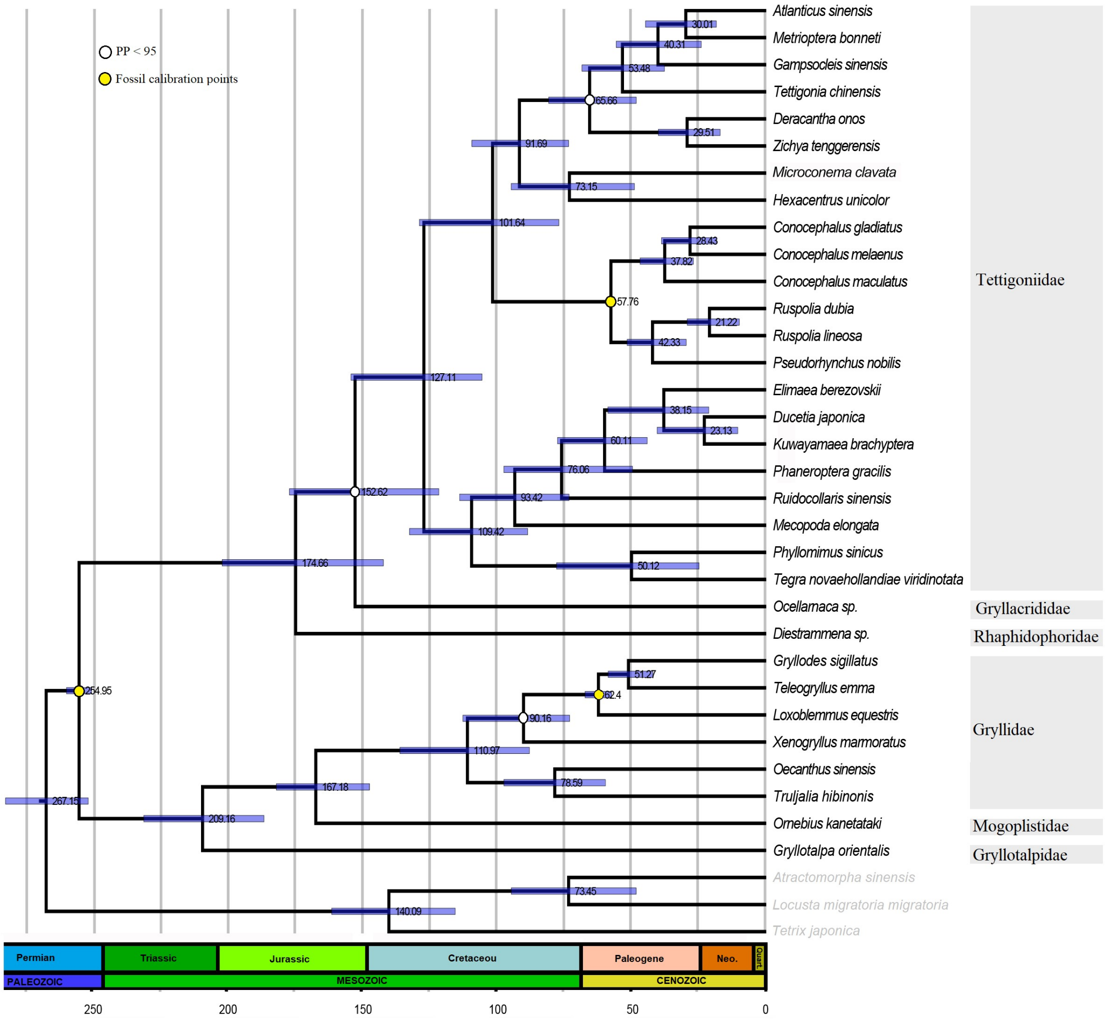

Figure S4 Ancestral reconstruction of genome size (male) of Ensifera based on a time-calibrated phylogenetic tree.

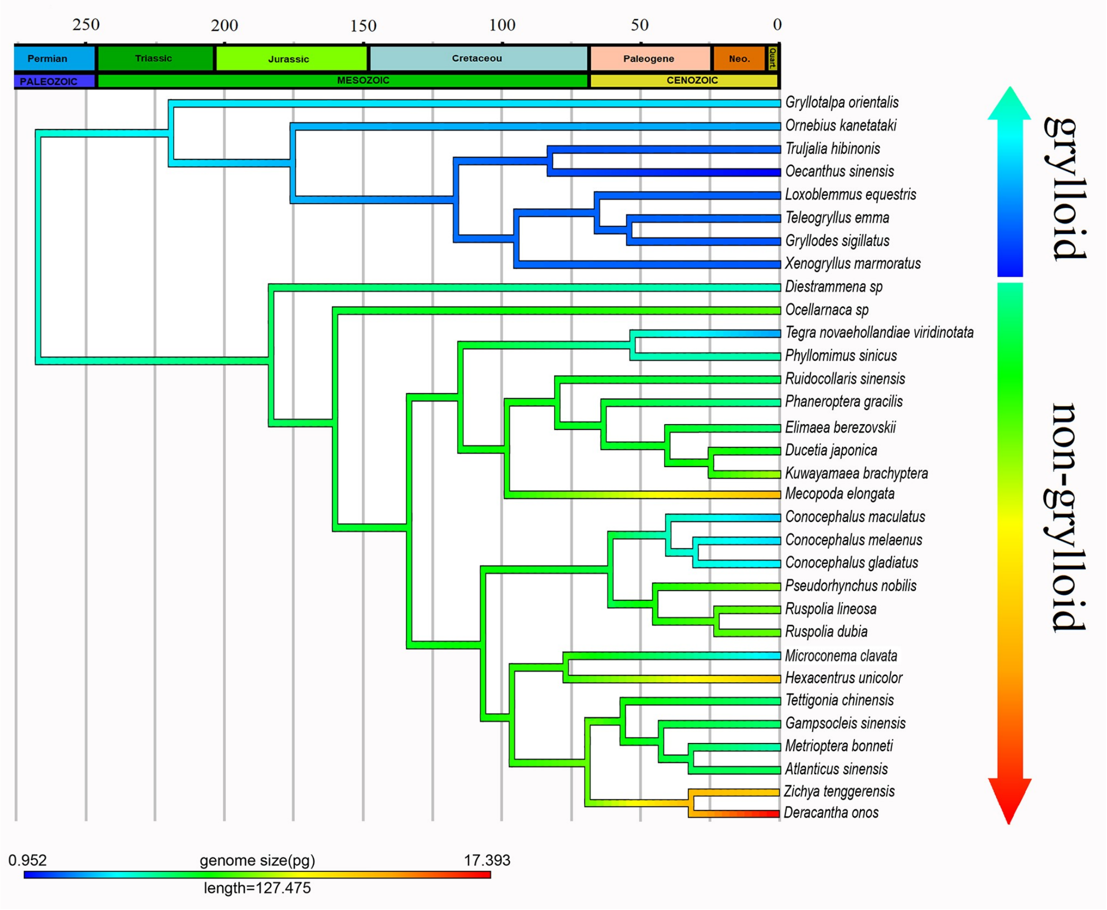

Supplement: Supplementary file 2 [file Data_Sheet_2.pdf]
