# Supplementary material for: The Evolutionary Patterns of Genome Size in Ensifera (Insecta: Orthoptera)
Source: Front Genet. 2021 Jun 23;12:693541. doi: 10.3389/fgene.2021.693541 (PMC8261143; doi:10.3389/fgene.2021.693541)
Supplement: Supplementary file 3 [file Image_1.PDF]

Additional file 2: Flow cytometry estimation of the genome size for the 32 Ensifera species.

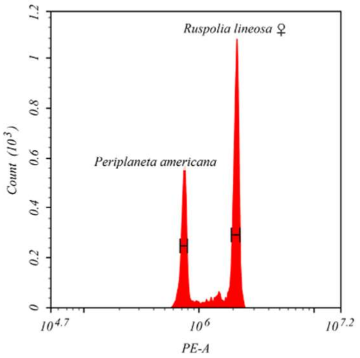

Species	Mean	CV	C-value
Periplaneta americana	750,954	3.68%	3.41
Ruspolia lineosa	2,135,913	3.98%	9.6989

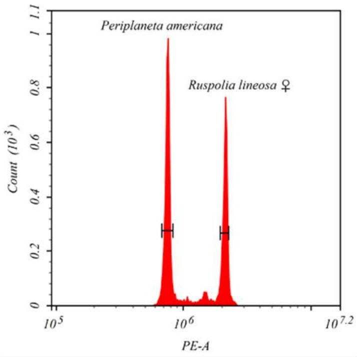

Species	Mean	CV	C-value
Periplaneta americana	761,234	3.60%	3.41
Ruspolia lineosa	2,164,841	3.23%	9.6976

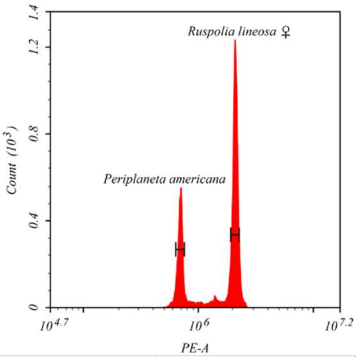

Species	Mean	CV	C-value
Periplaneta americana	704,229	3.89%	3.41
Ruspolia lineosa	2,121,233	3.99%	10.2714

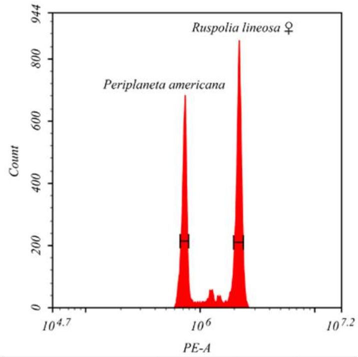

Species	Mean	CV	C-value
Periplaneta americana	736,670	3.76%	3.41
Ruspolia lineosa	2,207,859	4.32%	10.2200

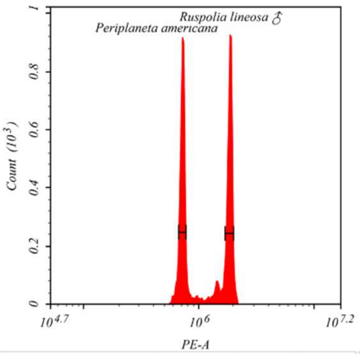

Species	Mean	CV	C-value
Periplaneta americana	729,693	3.69%	3.41
Ruspolia lineosa	1,884,980	3.89%	8.8089

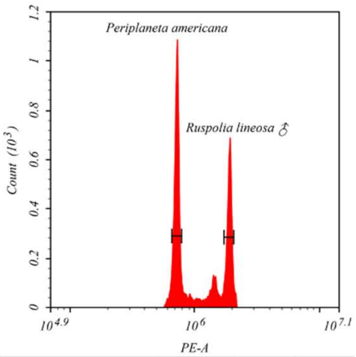

Species	Mean	CV	C-value
Periplaneta americana	728,076	3.57%	3.41
Ruspolia lineosa	1,922,534	3.58%	9.0013

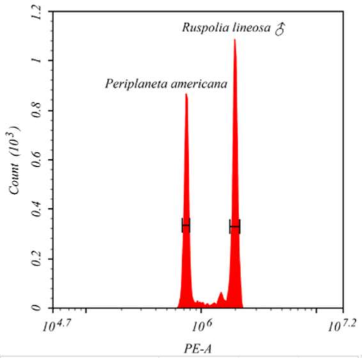

Species	Mean	CV	C-value
Periplaneta americana	751,950	3.51%	3.41
Ruspolia lineosa	1,993,888	3.88%	9.0420

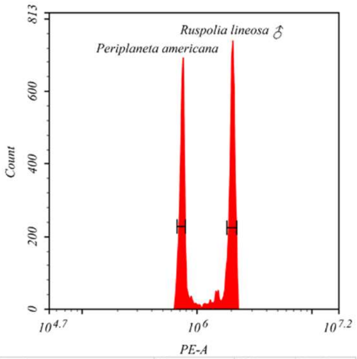

Species	Mean	CV	C-value
Periplaneta americana	749,035	3.81%	3.41
Ruspolia lineosa	2,048,650	4.16%	9.3265

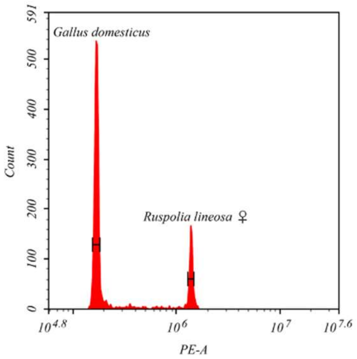

Species	Mean	CV	C-value
Gallus domesticus	170,494	3.45%	1.165
Ruspolia lineosa	1,397,814	2.78%	9.5514

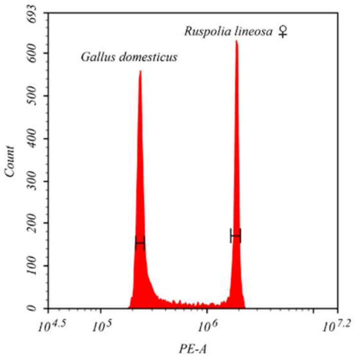

Species	Mean	CV	C-value
Gallus domesticus	234,732	4.47%	1.165
Ruspolia lineosa	1,920,038	3.88%	9.5294

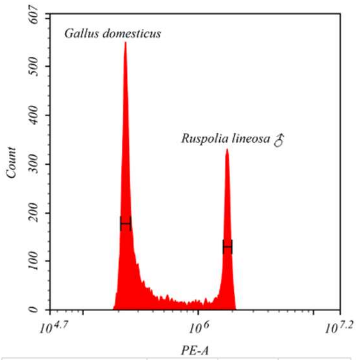

Species	Mean	CV	C-value
Gallus domesticus	236,163	4.94%	1.165
Ruspolia lineosa	1,812,839	3.92%	8.9428

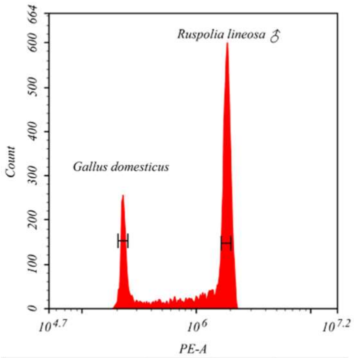

Species	Mean	CV	C-value
Gallus domesticus	231,914	4.02%	1.165
Ruspolia lineosa	1,873,137	4.94%	9.4095

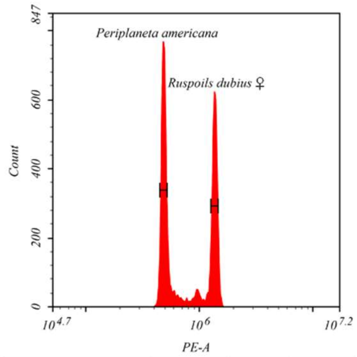

Species	Mean	CV	C-value
Periplaneta americana	491,732	3.58%	3.41
Ruspolia dubia	1,384,456	3.60%	9.6007

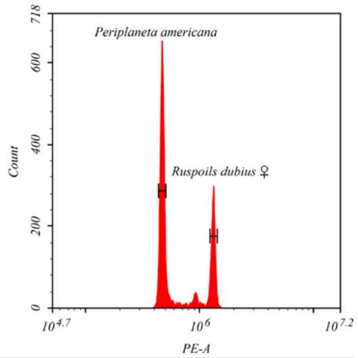

Species	Mean	CV	C-value
Periplaneta americana	477,098	3.27%	3.41
Ruspolia dubia	1,335,742	3.20%	9.5471

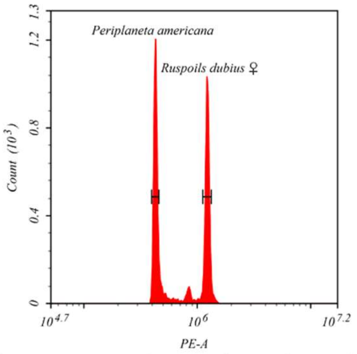

Species	Mean	CV	C-value
Periplaneta americana	433,099	3.01%	3.41
Ruspolia dubia	1,240,773	3.26%	9.7692

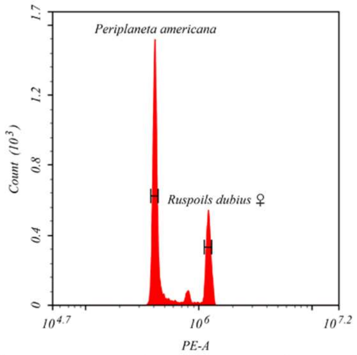

Species	Mean	CV	C-value
Periplaneta americana	414,658	3.23%	3.41
Ruspolia dubia	1,229,504	3.61%	10.1110

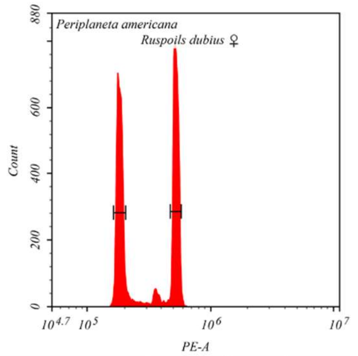

Species	Mean	CV	C-value
Periplaneta americana	184,763	4.52%	3.41
Ruspolia dubia	531,187	4.10%	9.8036

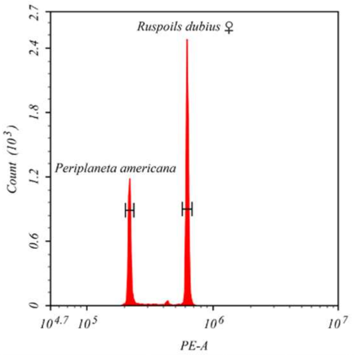

Species	Mean	CV	C-value
Periplaneta americana	220,314	2.25%	3.41
Ruspolia dubia	631,134	2.19%	9.7686

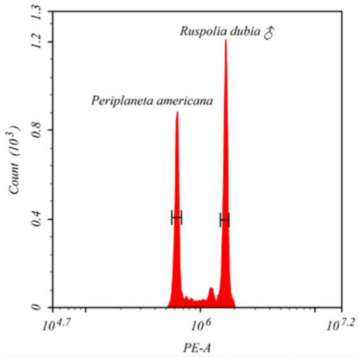

Species	Mean	CV	C-value
Periplaneta americana	630, 347	3. 57%	3.41
Ruspolia dubia	1, 675, 882	3. 53%	9.0661

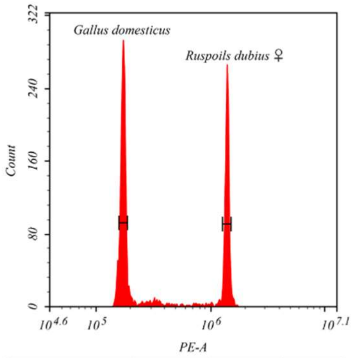

Species	Mean	CV	C-value
Gallus domesticus	174,782	3.62%	1.165
Ruspolia dubia	1,413,047	3.30%	9.4186

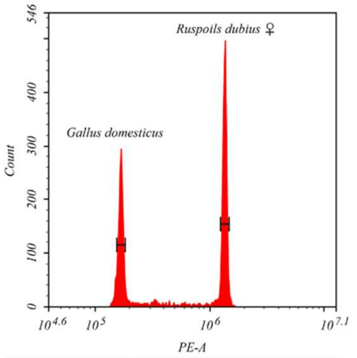

Species	Mean	CV	C-value
Gallus domesticus	169,966	3.59%	1.165
Ruspolia dubia	1,373,280	3.49%	9.4129

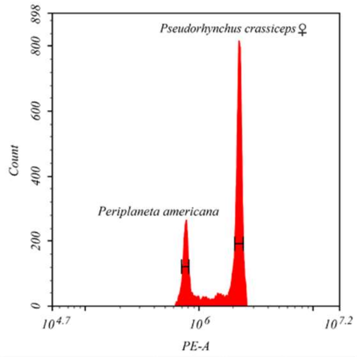

Species	Mean	CV	C-value
Periplaneta americana	770,163	3.72%	3.41
Pseudorhynchus crassiceps	2,271,062	3.89%	10.0554

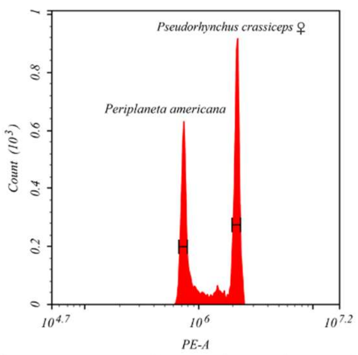

Species	Mean	CV	C-value
Periplaneta americana	740,238	3.87%	3.41
Pseudorhynchus crassiceps	2,181,416	3.90%	10.0490

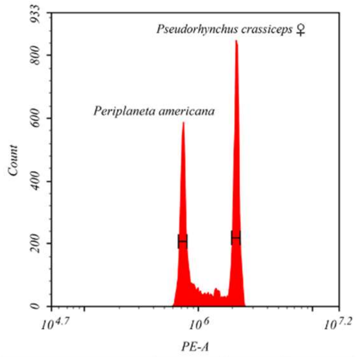

Species	Mean	CV	C-value
Periplaneta americana	738,437	3.87%	3.41
Pseudorhynchus crassiceps	2,173,427	3.65%	10.0366

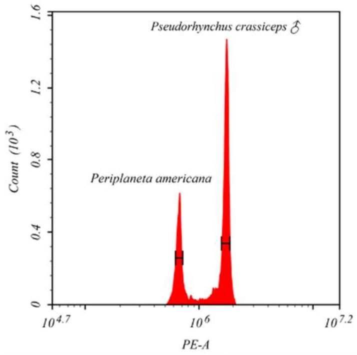

Species	Mean	CV	C-value
Periplaneta americana	671,890	3.70%	3.41
Pseudorhynchus crassiceps	1,756,055	3.74%	8.9124

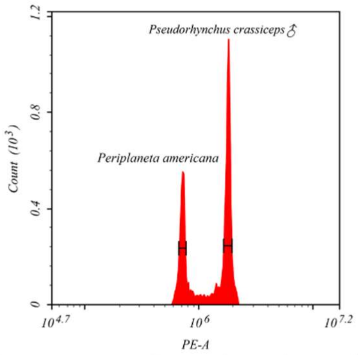

Species	Mean	CV	C-value
Periplaneta americana	725,524	3.70%	3.41
Pseudorhynchus crassiceps	1,824,077	3.80%	8.5733

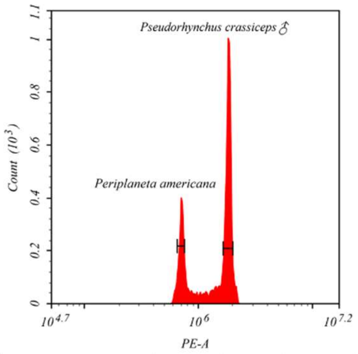

Species	Mean	CV	C-value
Periplaneta americana	716,449	3.72%	3.41
Pseudorhynchus crassiceps	1,855,454	4.00%	8.8312

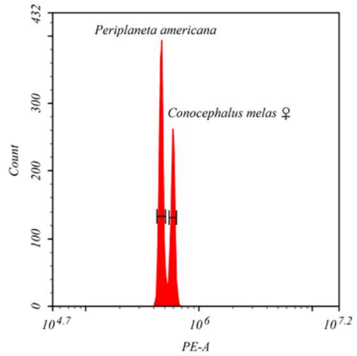

Species	Mean	CV	C-value
Periplaneta americana	469,939	3.30%	3.41
Conocephalus melaenus	597,939	3.24%	4.3388

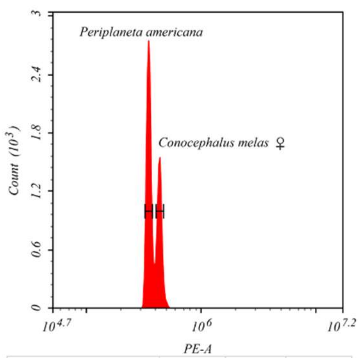

Species	Mean	CV	C-value
Periplaneta americana	354,207	3.47%	3.41
Conocephalus melaenus	438,540	3.43%	4.2219

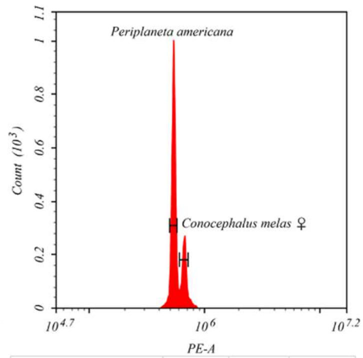

Species	Mean	CV	C-value
Periplaneta americana	545,255	3.19%	3.41
Conocephalus melaenus	672,741	3.67%	4.2073

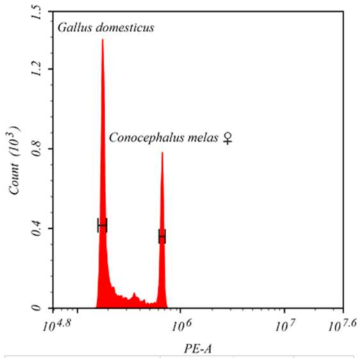

Species	Mean	CV	C-value
Gallus domesticus	178,914	3.79%	1.165
Conocephalus melaenus	666,973	2.89%	4.3430

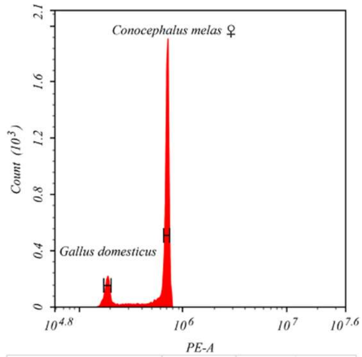

Species	Mean	CV	C-value
Gallus domesticus	189,088	3.96%	1.165
Conocephalus melaenus	713,268	3.12%	4.3946

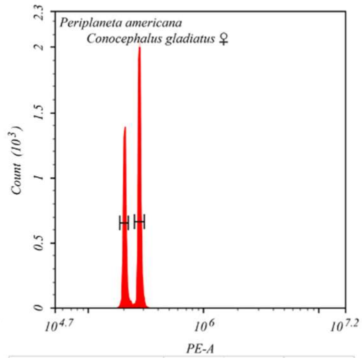

Species	Mean	CV	C-value
Periplaneta americana	209,177	2.58%	3.41
Conocephalus gladiatus	281,653	2.56%	4.5915

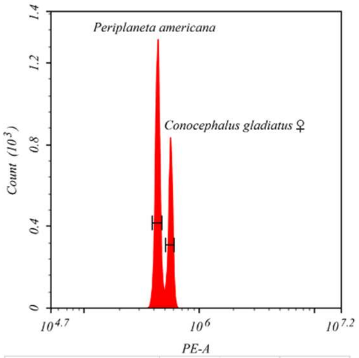

Species	Mean	CV	C-value
Periplaneta americana	439,284	3.78%	3.41
Conocephalus gladiatus	570,295	3.52%	4.4270

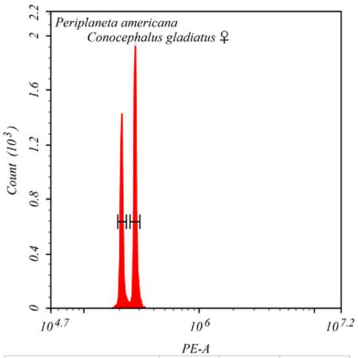

Species	Mean	CV	C-value
Periplaneta americana	214,640	2.70%	3.41
Conocephalus gladiatus	281,418	2.61%	4.4709

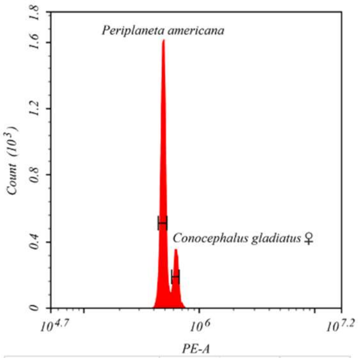

Species	Mean	CV	C-value
Periplaneta americana	490,185	3.75%	3.41
Conocephalus gladiatus	632,689	3.84%	4.4013

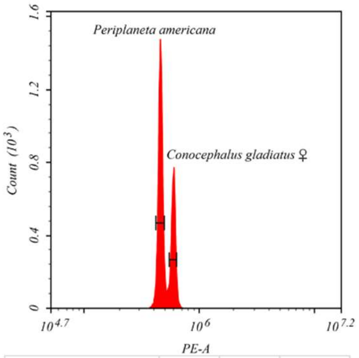

Species	Mean	CV	C-value
Periplaneta americana	466,012	3.65%	3.41
Conocephalus gladiatus	602,297	3.50%	4.4073

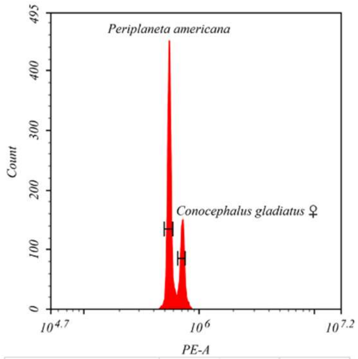

Species	Mean	CV	C-value
Periplaneta americana	555,955	2.96%	3.41
Conocephalus gladiatus	721,304	3.34%	4.4242

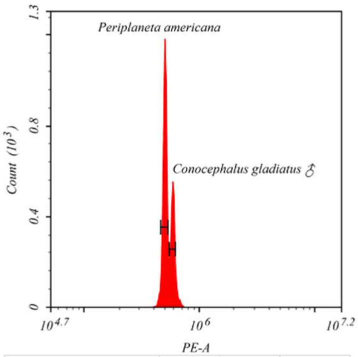

Species	Mean	CV	C-value
Periplaneta americana	509,605	3.21%	3.41
Conocephalus gladiatus	592,513	2.81%	3.9648

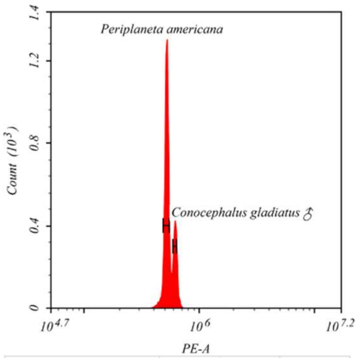

Species	Mean	CV	C-value
Periplaneta americana	526,933	2.75%	3.41
Conocephalus gladiatus	620,711	2.19%	4.0169

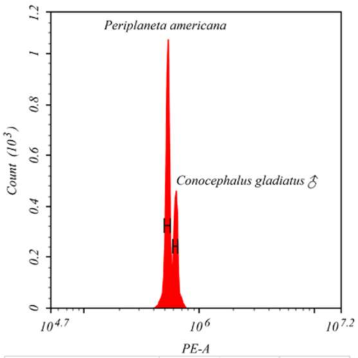

Species	Mean	CV	C-value
Periplaneta americana	537,272	2.79%	3.41
Conocephalus gladiatus	630,316	2.18%	4.0005

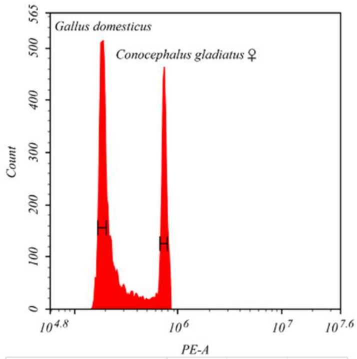

Species	Mean	CV	C-value
Gallus domesticus	187,613	4.86%	1.165
Conocephalus gladiatus	740,823	3.81%	4.6002

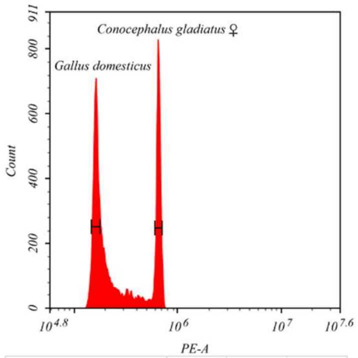

Species	Mean	CV	C-value
Gallus domesticus	164,020	4.65%	1.165
Conocephalus gladiatus	660,385	3.44%	4.6906

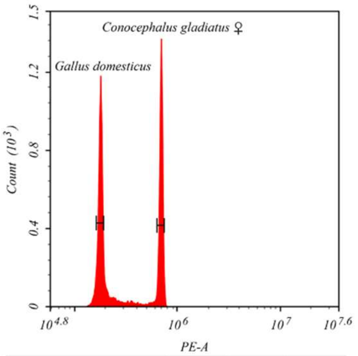

Species	Mean	CV	C-value
Gallus domesticus	180,846	3.70%	1.165
Conocephalus gladiatus	704,539	2.91%	4.5386

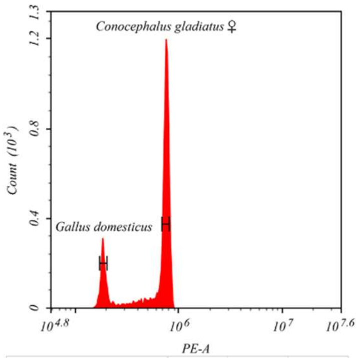

Species	Mean	CV	C-value
Gallus domesticus	183,642	4.13%	1.165
Conocephalus gladiatus	766,414	4.15%	4.8620

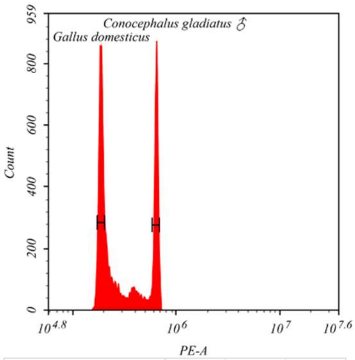

Species	Mean	CV	C-value
Gallus domesticus	189,923	3.92%	1.165
Conocephalus gladiatus	650,505	3.41%	3.9902

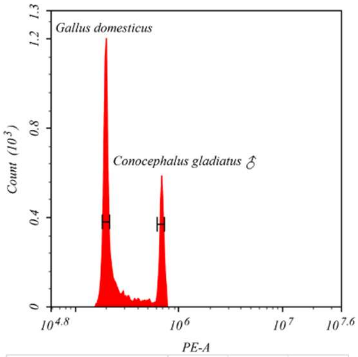

Species	Mean	CV	C-value
Gallus domesticus	198,870	3.81%	1.165
Conocephalus gladiatus	688,974	3.45%	4.0361

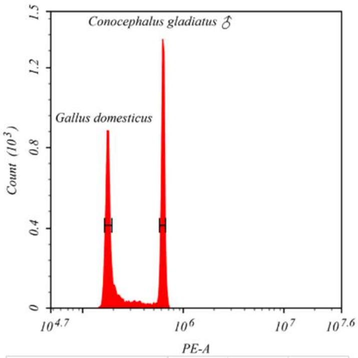

Species	Mean	CV	C-value
Gallus domesticus	178,491	3.86%	1.165
Conocephalus gladiatus	632,681	3.03%	4.1295

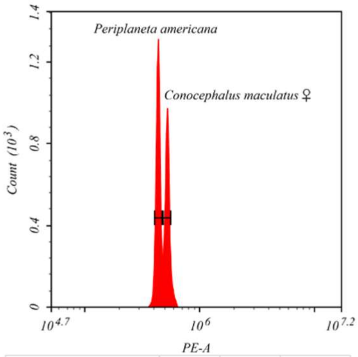

Species	Mean	CV	C-value
Periplaneta americana	441,205	3.17%	3.41
Conocephalus maculatus	528,347	3.48%	4.0835

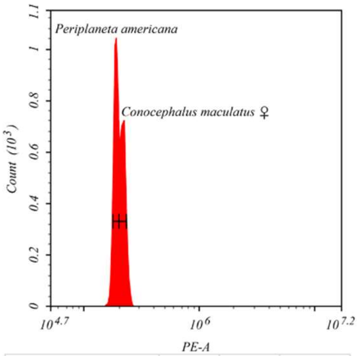

Species	Mean	CV	C-value
Periplaneta americana	191,727	3.53%	3.41
Conocephalus maculatus	218,902	3.79%	3.8933

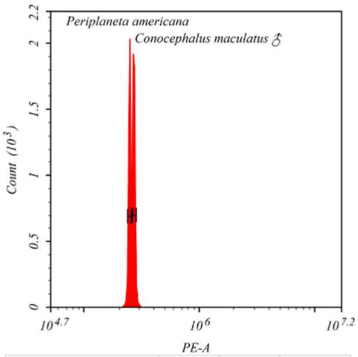

Species	Mean	CV	C-value
Periplaneta americana	251,490	1.69%	3.41
Conocephalus maculatus	273,483	1.69%	3.7082

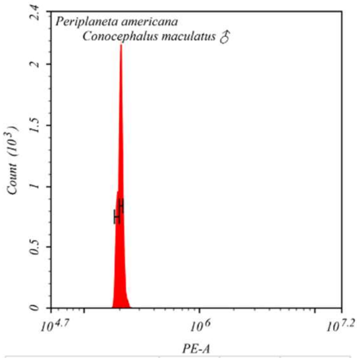

Species	Mean	CV	C-value
Periplaneta americana	194,652	1.99%	3.41
Conocephalus maculatus	209,277	1.90%	3.6662

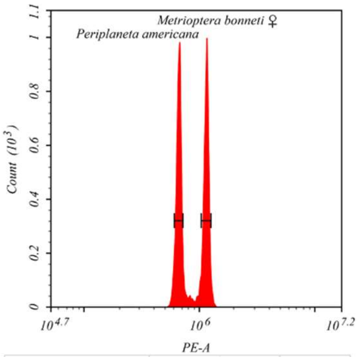

Species	Mean	CV	C-value
Periplaneta americana	670,549	4.09%	3.41
Metrioptera bonneti	1,162,057	4.20%	5.9095

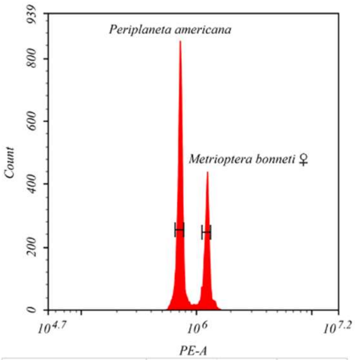

Species	Mean	CV	C-value
Periplaneta americana	728,451	3.84%	3.41
Metrioptera bonneti	1,246,434	3.86%	5.8348

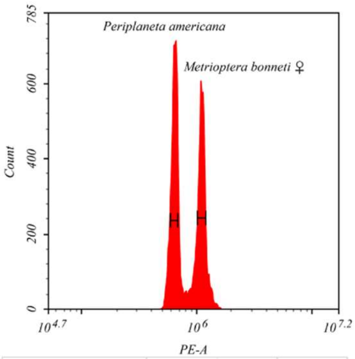

Species	Mean	CV	C-value
Periplaneta americana	650,629	4.01%	3.41
Metrioptera bonneti	1,113,646	4.23%	5.8367

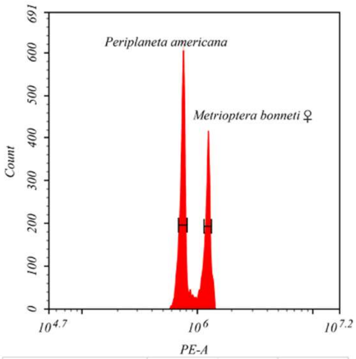

Species	Mean	CV	C-value
Periplaneta americana	754,604	3.79%	3.41
Metrioptera bonneti	1,244,026	3.64%	5.6217

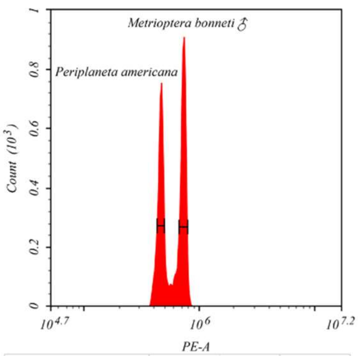

Species	Mean	CV	C-value
Periplaneta americana	469,000	3.78%	3.41
Metrioptera bonneti	736,859	4.00%	5.3575

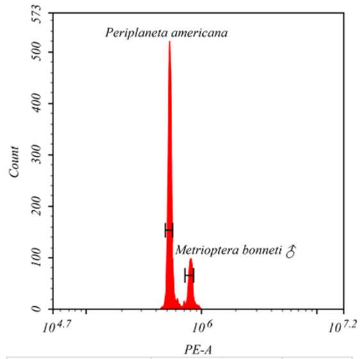

Species	Mean	CV	C-value
Periplaneta americana	535,572	2.83%	3.41
Metrioptera bonneti	810,918	3.37%	5.1631

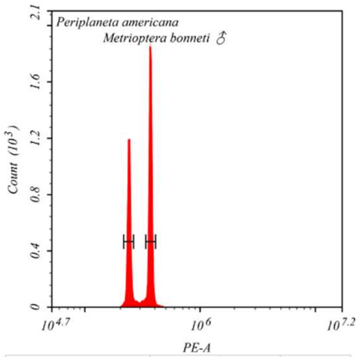

Species	Mean	CV	C-value
Periplaneta americana	244,273	2.85%	3.41
Metrioptera bonneti	374,997	2.77%	5.2349

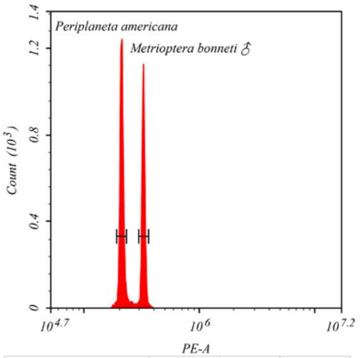

Species	Mean	CV	C-value
Periplaneta americana	214,879	3.20%	3.41
Metrioptera bonneti	331,403	2.80%	5.2592

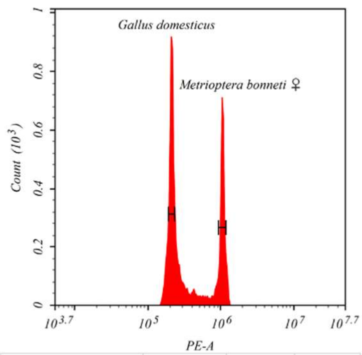

Species	Mean	CV	C-value
Gallus domesticus	214,434	4.91%	1.165
Metrioptera bonneti	1,067,581	4.50%	5.8001

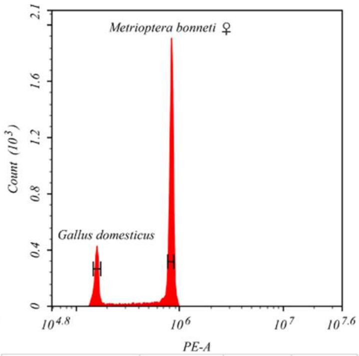

Species	Mean	CV	C-value
Gallus domesticus	159,587	3.52%	1.165
Metrioptera bonneti	840,810	3.15%	6.1380

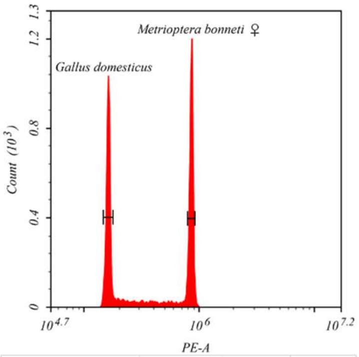

Species	Mean	CV	C-value
Gallus domesticus	163,689	3.50%	1.165
Metrioptera bonneti	863,865	2.99%	6.1483

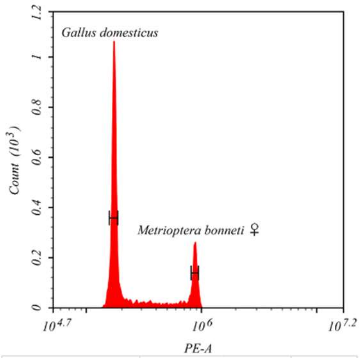

Species	Mean	CV	C-value
Gallus domesticus	175,425	3.56%	1.165
Metrioptera bonneti	885,061	3.19%	5.8777

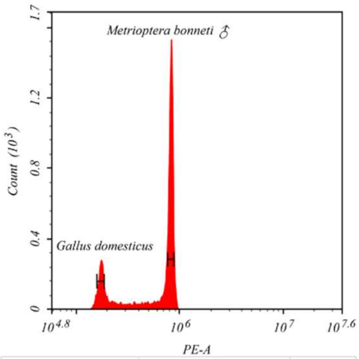

Species	Mean	CV	C-value
Gallus domesticus	175,912	4.03%	1.165
Metrioptera bonneti	835,242	3.36%	5.5315

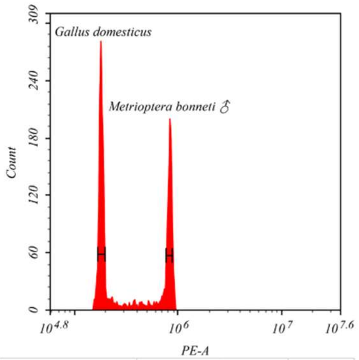

Species	Mean	CV	C-value
Gallus domesticus	183,131	3.66%	1.165
Metrioptera bonneti	841,764	3.55%	5.3549

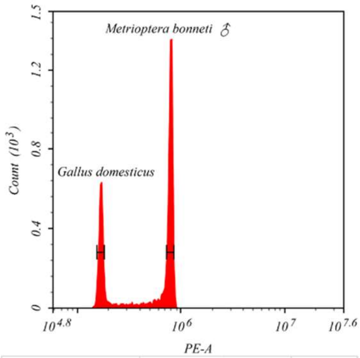

Species	Mean	CV	C-value
Gallus domesticus	170,615	3.65%	1.165
Metrioptera bonneti	805,149	3.38%	5.4977

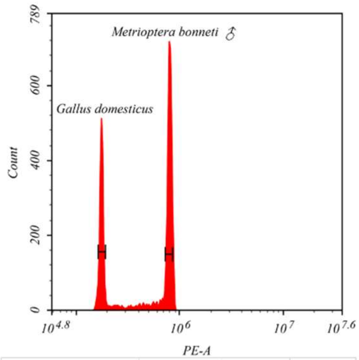

Species	Mean	CV	C-value
Gallus domesticus	177,953	3.28%	1.165
Metrioptera bonneti	811,650	3.71%	5.3136

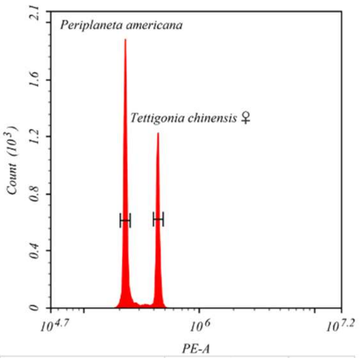

Species	Mean	CV	C-value
Periplaneta americana	230,933	3.03%	3.41
Tettigonia chinensis Willemse	443,239	2.98%	6.5450

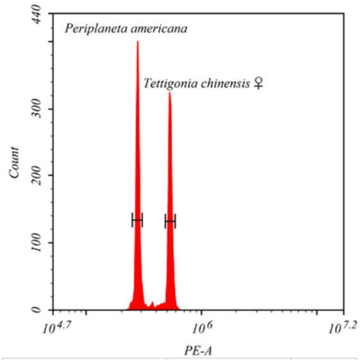

Species	Mean	CV	C-value
Periplaneta americana	283,322	3.24%	3.41
Tettigonia chinensis Willemse	541,639	3.36%	6.5190

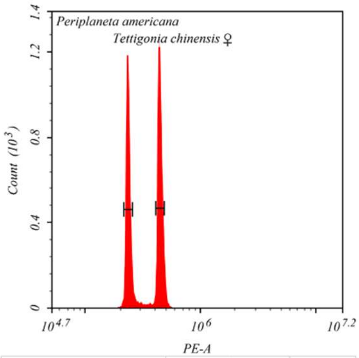

Species	Mean	CV	C-value
Periplaneta americana	238,635	3.19%	3.41
Tettigonia chinensis Willemse	452,012	3.62%	6.4591

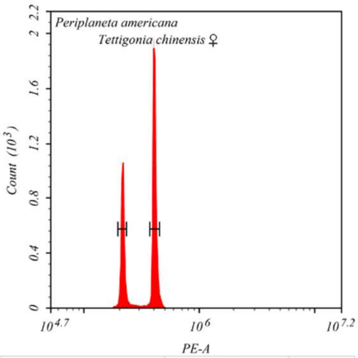

Species	Mean	CV	C-value
Periplaneta americana	219,194	2.97%	3.41
Tettigonia chinensis Willemse	414,108	2.96%	6.4423

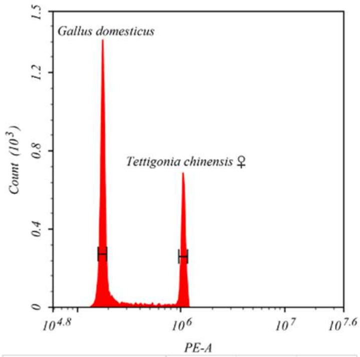

Species	Mean	CV	C-value
Gallus domesticus	177,662	3.96%	1.165
Tettigonia chinensis Willemse	1,066,781	3.80%	6.9953

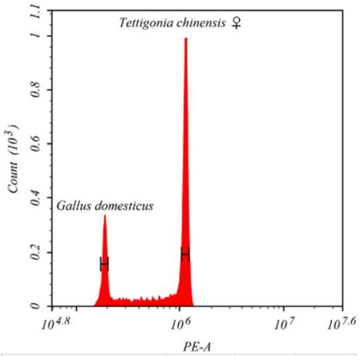

Species	Mean	CV	C-value
Gallus domesticus	190,727	3.67%	1.165
Tettigonia chinensis Willemse	1,146,365	3.70%	7.0022

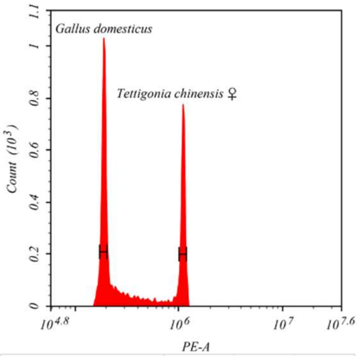

Species	Mean	CV	C-value
Gallus domesticus	191,582	3.64%	1.165
Tettigonia chinensis Willemse	1,117,628	3.42%	6.7962

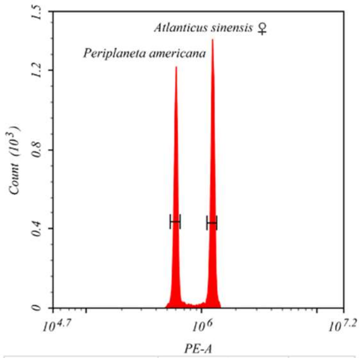

Species	Mean	CV	C-value
Periplaneta americana	604,994	3.28%	3.41
Atlanticus sinensis Uvarov	1,262,388	3.46%	7.1153

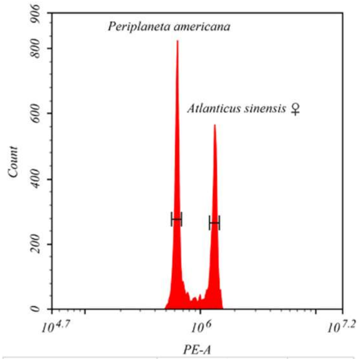

Species	Mean	CV	C-value
Periplaneta americana	632,524	3.86%	3.41
Atlanticus sinensis Uvarov	1,340,540	4.15%	7.2270

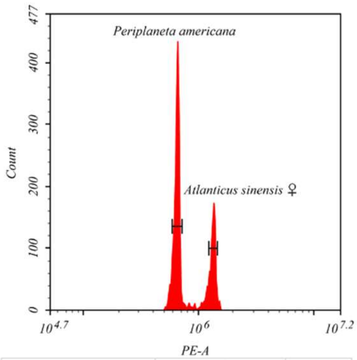

Species	Mean	CV	C-value
Periplaneta americana	657,420	4.19%	3.41
Atlanticus sinensis Uvarov	1,357,391	3.89%	7.0407

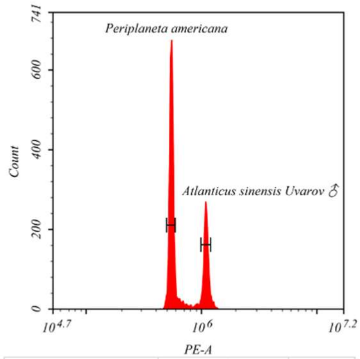

Species	Mean	CV	C-value
Periplaneta americana	553,470	3.15%	3.41
Atlanticus sinensis Uvarov	1,105,738	3.79%	6.8126

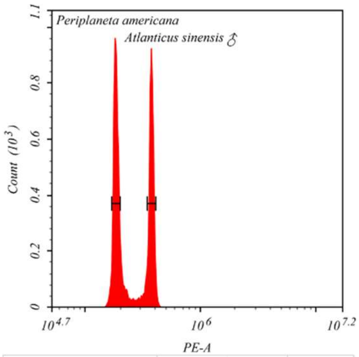

Species	Mean	CV	C-value
Periplaneta americana	187,427	3.90%	3.41
Atlanticus sinensis Uvarov	380,609	3.73%	6.9247

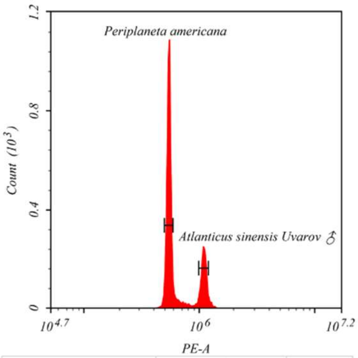

Species	Mean	CV	C-value
Periplaneta americana	550,439	3.34%	3.41
Atlanticus sinensis Uvarov	1,107,239	4.28%	6.8594

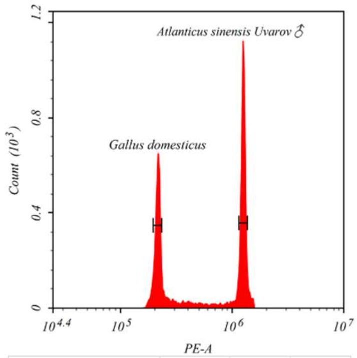

Species	Mean	CV	C-value
Gallus domesticus	216,820	4.17%	1.165
Atlanticus sinensis Uvarov	1,259,947	3.79%	6.7698

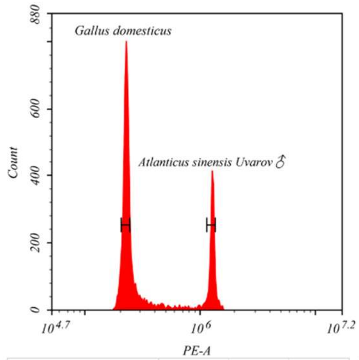

Species	Mean	CV	C-value
Gallus domesticus	229,627	4.26%	1.165
Atlanticus sinensis Uvarov	1,287,915	3.70%	6.5342

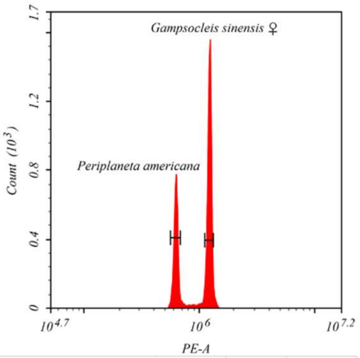

Species	Mean	CV	C-value
Periplaneta americana	633,603	3.61%	3.41
Gampsocleis sinensis	1,241,981	3.52%	6.6842

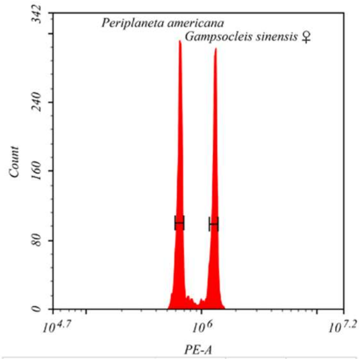

Species	Mean	CV	C-value
Periplaneta americana	654,794	4.05%	3.41
Gampsocleis sinensis	1,313,631	3.88%	6.8411

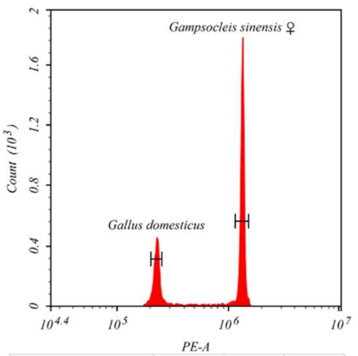

Species	Mean	CV	C-value
Gallus domesticus	231,065	4.60%	1.165
Gampsocleis sinensis	1,362,260	3.72%	6.8683

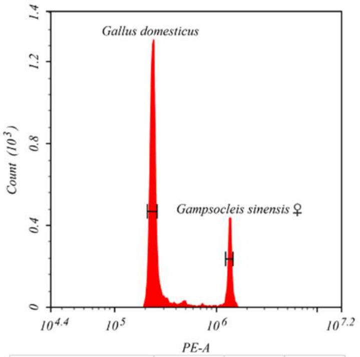

Species	Mean	CV	C-value
Gallus domesticus	239,471	4.61%	1.165
Gampsocleis sinensis	1,385,788	3.81%	6.7417

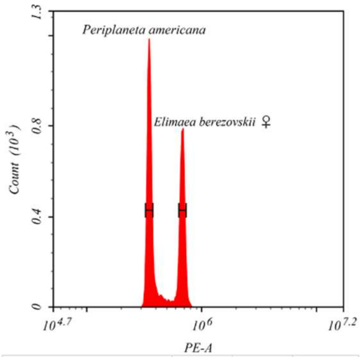

Species	Mean	CV	C-value
Periplaneta americana	354,930	3.33%	3.41
Elimaea berezovskii Bey-Bienko	688,533	3.51%	6.6151

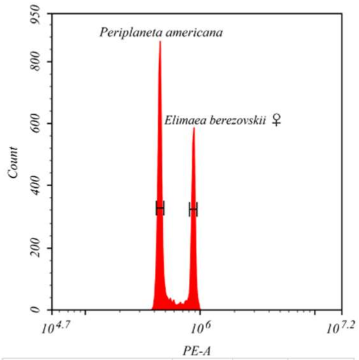

Species	Mean	CV	C-value
Periplaneta americana	450,714	3.11%	3.41
Elimaea berezovskii Bey-Bienko	884,461	2.97%	6.6916

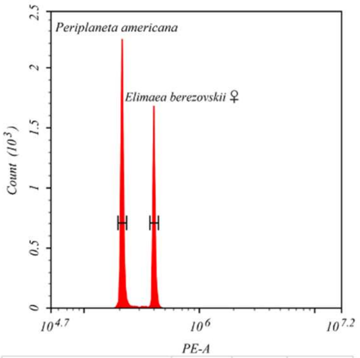

Species	Mean	CV	C-value
Periplaneta americana	215,848	2.53%	3.41
Elimaea berezovskii Bey-Bienko	408,557	2.34%	6.4544

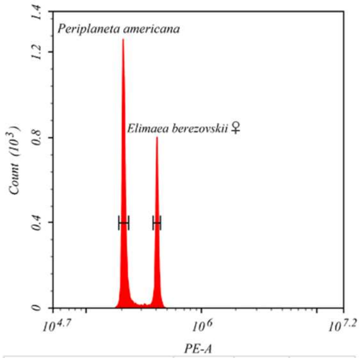

Species	Mean	CV	C-value
Periplaneta americana	214,397	3.24%	3.41
Elimaea berezovskii Bey-Bienko	416,236	2.62%	6.6203

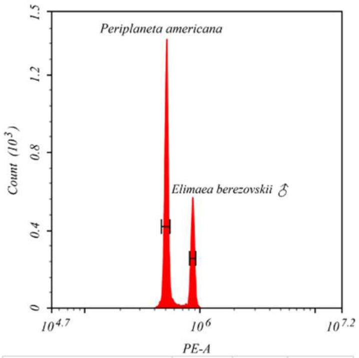

Species	Mean	CV	C-value
Periplaneta americana	516,469	2.96%	3.41
Elimaea berezovskii Bey-Bienko	874,356	2.76%	5.7730

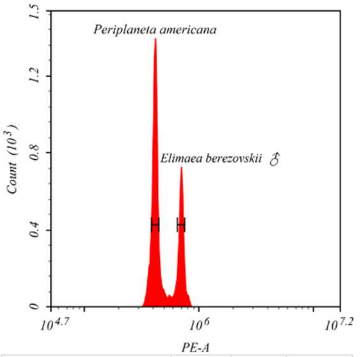

Species	Mean	CV	C-value
Periplaneta americana	421,082	3.50%	3.41
Elimaea berezovskii Bey-Bienko	712,167	3.45%	5.7673

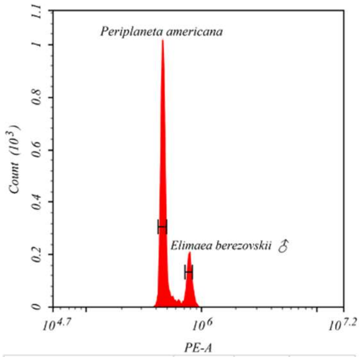

Species	Mean	CV	C-value
Periplaneta americana	467,207	3.47%	3.41
Elimaea berezovskii Bey-Bienko	791,514	3.29%	5.7770

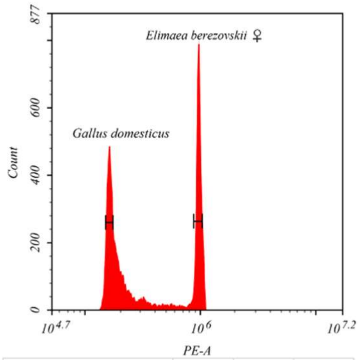

Species	Mean	CV	C-value
Gallus domesticus	163,339	3.55%	1.165
Elimaea berezovskii Bey-Bienko	979,490	3.55%	6.9861

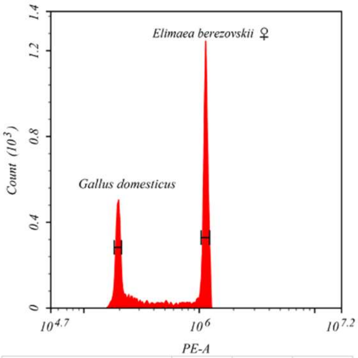

Species	Mean	CV	C-value
Gallus domesticus	199,558	3.35%	1.165
Elimaea berezovskii Bey-Bienko	1,150,217	3.58%	6.7149

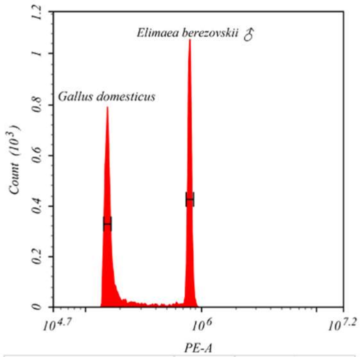

Species	Mean	CV	C-value
Gallus domesticus	154,375	3.61%	1.165
Elimaea berezovskii Bey-Bienko	806,073	2.97%	6.0831

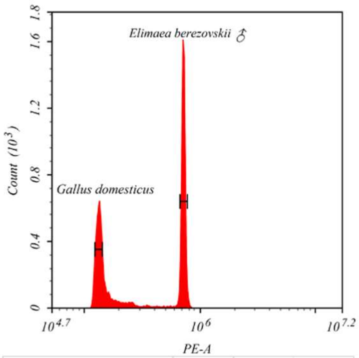

Species	Mean	CV	C-value
Gallus domesticus	134,455	4.10%	1.165
Elimaea berezovskii Bey-Bienko	728,357	2.90%	6.3109

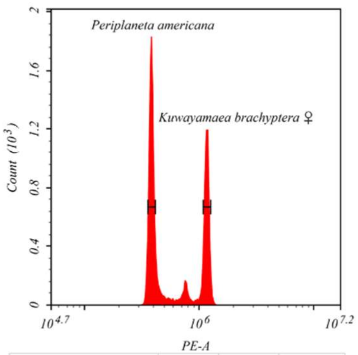

Species	Mean	CV	C-value
Periplaneta americana	388,107	3.45%	3.41
Kuwayamaea brachyptera	1,181,836	3.45%	10.3839

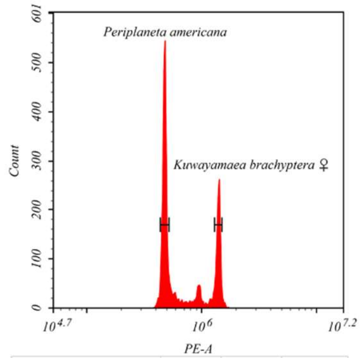

Species	Mean	CV	C-value
Periplaneta americana	482,166	3.11%	3.41
Kuwayamaea brachyptera	1,423,558	3.01%	10.0678

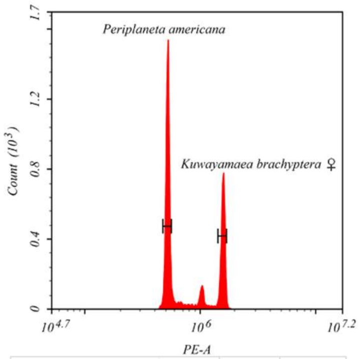

Species	Mean	CV	C-value
Periplaneta americana	527,687	3.10%	3.41
Kuwayamaea brachyptera	1,594,808	3.30%	10.3059

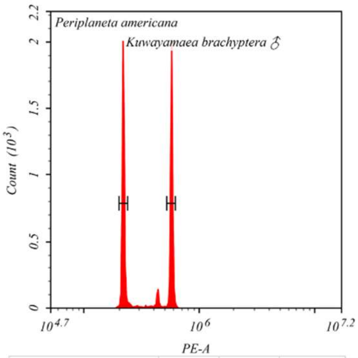

Species	Mean	CV	C-value
Periplaneta americana	222,057	2.18%	3.41
Kuwayamaea brachyptera	582,519	2.18%	8.9454

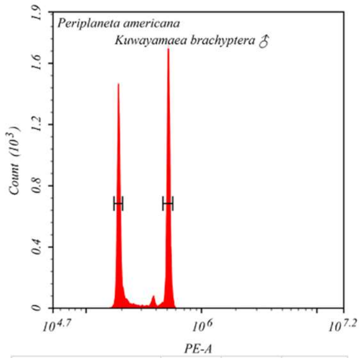

Species	Mean	CV	C-value
Periplaneta americana	193,610	2.71%	3.41
Kuwayamaea brachyptera	523,462	2.79%	9.2196

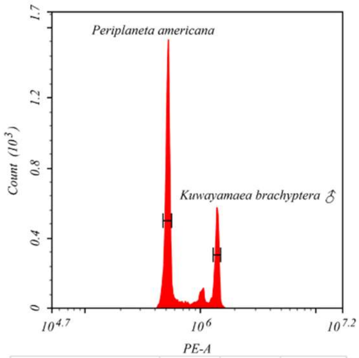

Species	Mean	CV	C-value
Periplaneta americana	525,652	3.47%	3.41
Kuwayamaea brachyptera	1,408,056	3.24%	9.1343

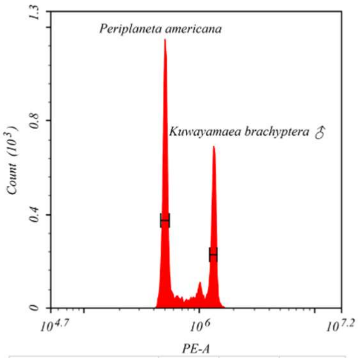

Species	Mean	CV	C-value
Periplaneta americana	510,786	3.46%	3.41
Kuwayamaea brachyptera	1,348,713	3.56%	9.0040

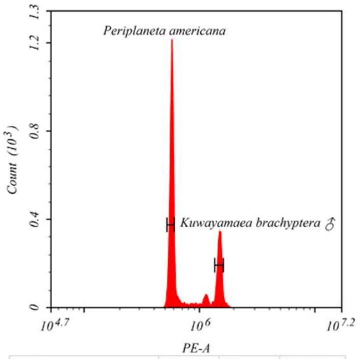

Species	Mean	CV	C-value
Periplaneta americana	579,704	3.06%	3.41
Kuwayamaea brachyptera	1,510,165	3.52%	8.8833

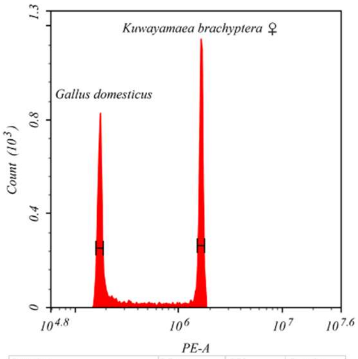

Species	Mean	CV	C-value
Gallus domesticus	175,284	3.75%	1.165
Kuwayamaea brachyptera	1,667,909	3.39%	11.0855

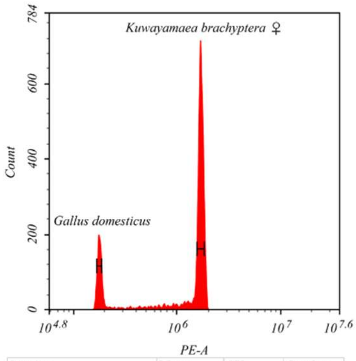

Species	Mean	CV	C-value
Gallus domesticus	179,264	3.20%	1.165
Kuwayamaea brachyptera	1,713,857	3.92%	11.1380

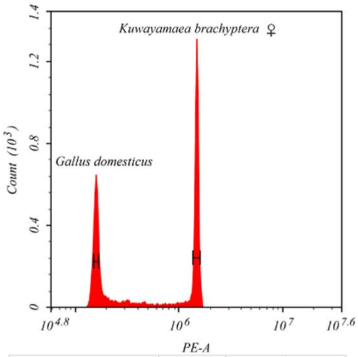

Species	Mean	CV	C-value
Gallus domesticus	160,056	3.30%	1.165
Kuwayamaea brachyptera	1,499,319	3.20%	10.9131

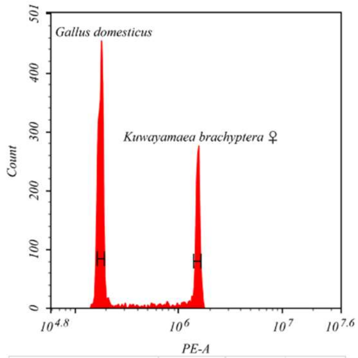

Species	Mean	CV	C-value
Gallus domesticus	178,074	4.35%	1.165
Kuwayamaea brachyptera	1,550,790	3.42%	10.1456

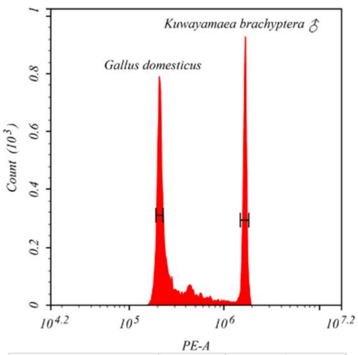

Species	Mean	CV	C-value
Gallus domesticus	213,928	3.93%	1.165
Kuwayamaea brachyptera	1,688,266	3.78%	9.1939

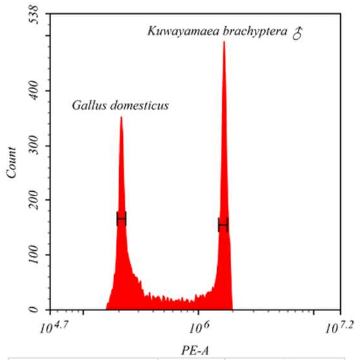

Species	Mean	CV	C-value
Gallus domesticus	216,105	4.55%	1.165
Kuwayamaea brachyptera	1,673,105	4.38%	9.0195

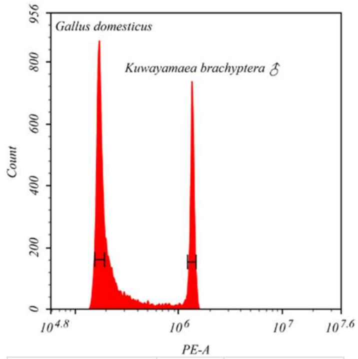

Species	Mean	CV	C-value
Gallus domesticus	173,443	4.89%	1.165
Kuwayamaea brachyptera	1,367,038	3.74%	9.1823

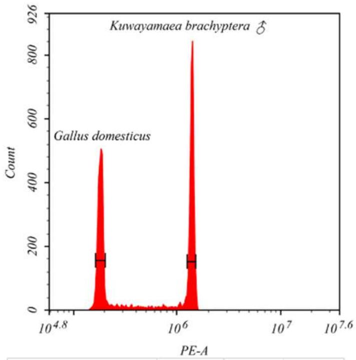

Species	Mean	CV	C-value
Gallus domesticus	184,080	4.46%	1.165
Kuwayamaea brachyptera	1,408,170	3.69%	8.9120

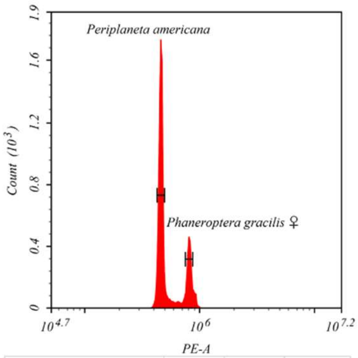

Species	Mean	CV	C-value
Periplaneta americana	465,335	3.11%	3.41
Phaneroptera gracilis	820,913	3.31%	6.0157

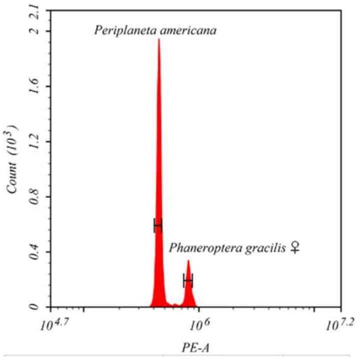

Species	Mean	CV	C-value
Periplaneta americana	453,446	3.14%	3.41
Phaneroptera gracilis	817,627	3.57%	6.1487

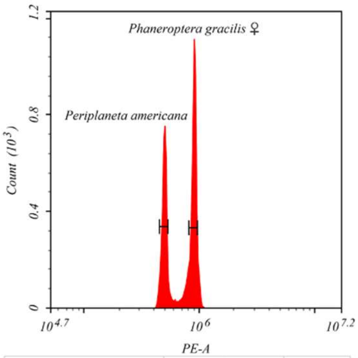

Species	Mean	CV	C-value
Periplaneta americana	503,009	3.77%	3.41
Phaneroptera gracilis	910,202	3.77%	6.1704

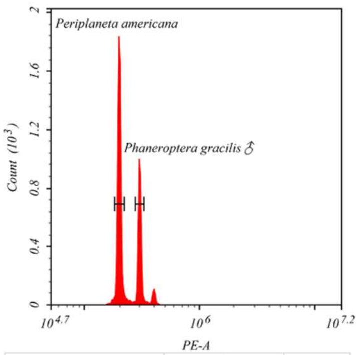

Species	Mean	CV	C-value
Periplaneta americana	203,126	2.99%	3.41
Phaneroptera gracilis	305,494	2.66%	5.1285

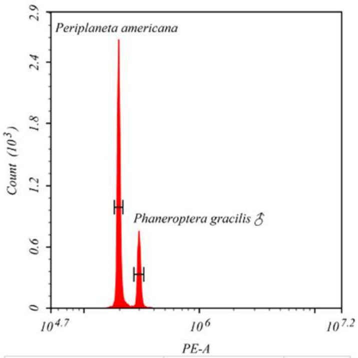

Species	Mean	CV	C-value
Periplaneta americana	201,714	2.59%	3.41
Phaneroptera gracilis	303,233	2.85%	5.1262

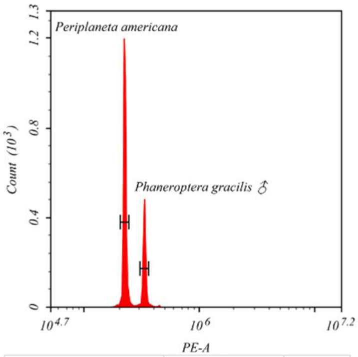

Species	Mean	CV	C-value
Periplaneta americana	228,116	2.38%	3.41
Phaneroptera gracilis	338,079	2.55%	5.0538

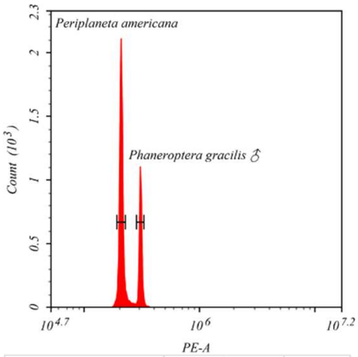

Species	Mean	CV	C-value
Periplaneta americana	211,279	2.92%	3.41
Phaneroptera gracilis	311,836	2.34%	5.0330

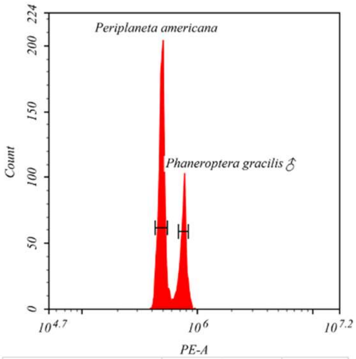

Species	Mean	CV	C-value
Periplaneta americana	494,835	4.80%	3.41
Phaneroptera gracilis	766,323	4.39%	5.2809

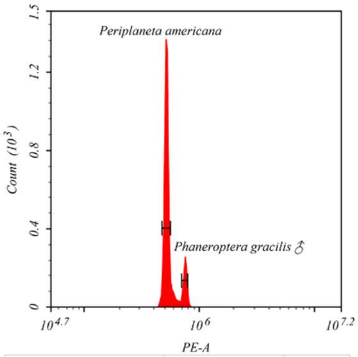

Species	Mean	CV	C-value
Periplaneta americana	523,605	3.36%	3.41
Phaneroptera gracilis	762,166	3.13%	4.9636

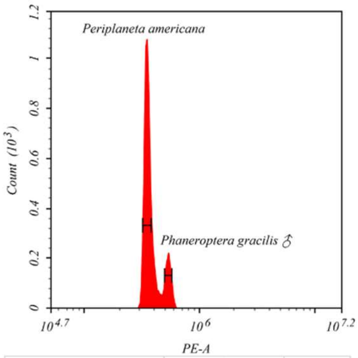

Species	Mean	CV	C-value
Periplaneta americana	353,524	4.26%	3.41
Phaneroptera gracilis	541,285	3.69%	5.2211

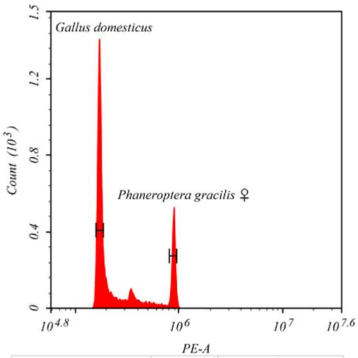

Species	Mean	CV	C-value
Gallus domesticus	173,324	3.48%	1.165
Phaneroptera gracilis	902,065	3.07%	6.0632

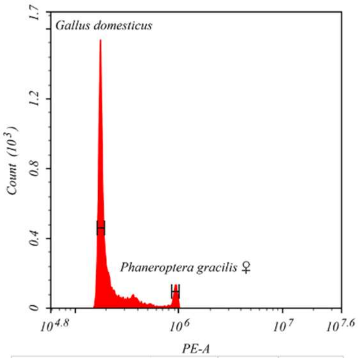

Species	Mean	CV	C-value
Gallus domesticus	177,632	3.59%	1.165
Phaneroptera gracilis	940,340	3.57%	6.1672

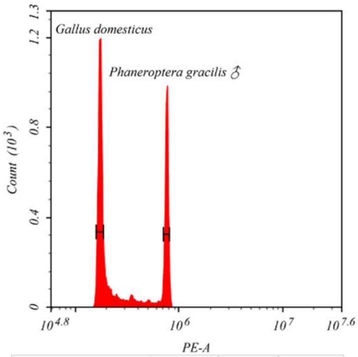

Species	Mean	CV	C-value
Gallus domesticus	175,707	3.43%	1.165
Phaneroptera gracilis	771,315	2.99%	5.1141

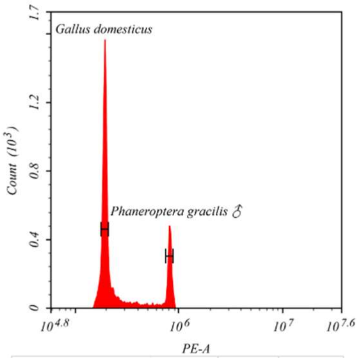

Species	Mean	CV	C-value
Gallus domesticus	195,044	3.51%	1.165
Phaneroptera gracilis	826,978	3.26%	4.9395

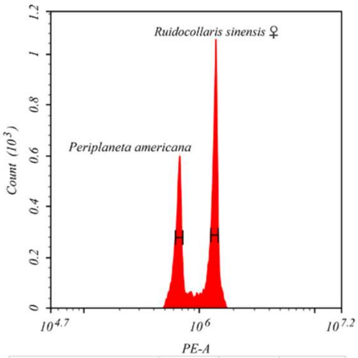

Species	Mean	CV	C-value
Periplaneta americana	671,935	3.89%	3.41
Ruidocollaris sinensis	1,385,646	3.72%	7.0320

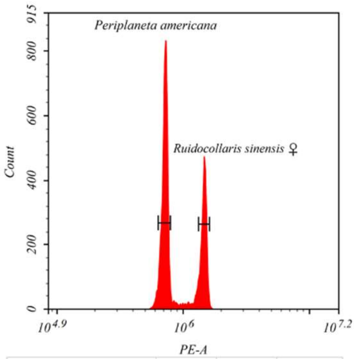

Species	Mean	CV	C-value
Periplaneta americana	722,085	4.44%	3.41
Ruidocollaris sinensis	1,476,171	4.04%	6.9711

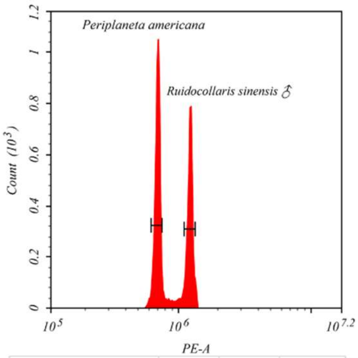

Species	Mean	CV	C-value
Periplaneta americana	707,875	3.78%	3.41
Ruidocollaris sinensis	1,243,421	3.69%	5.9899

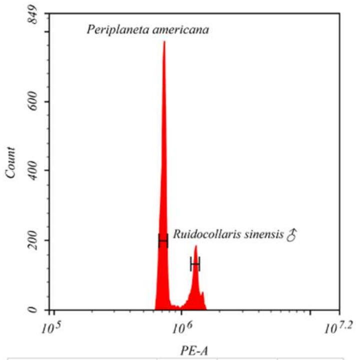

Species	Mean	CV	C-value
Periplaneta americana	728,279	3.62%	3.41
Ruidocollaris sinensis	1,294,650	3.86%	6.0619

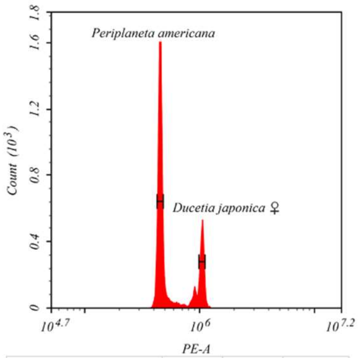

Species	Mean	CV	C-value
Periplaneta americana	459,359	3.04%	3.41
Ducetia japonica	1,064,684	3.14%	7.9036

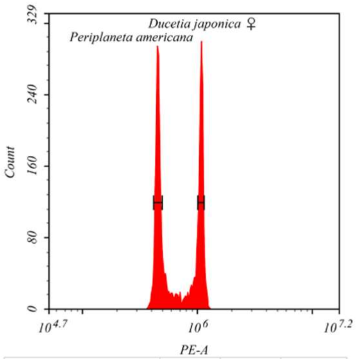

Species	Mean	CV	C-value
Periplaneta americana	458,874	3.51%	3.41
Ducetia japonica	1,093,905	3.04%	8.1291

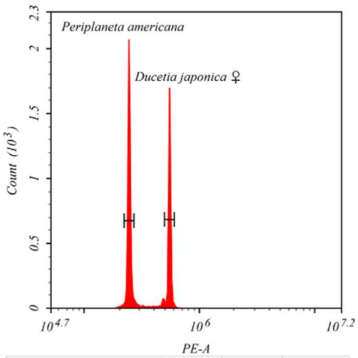

Species	Mean	CV	C-value
Periplaneta americana	248,382	2.65%	3.41
Ducetia japonica	557,598	2.60%	7.6552

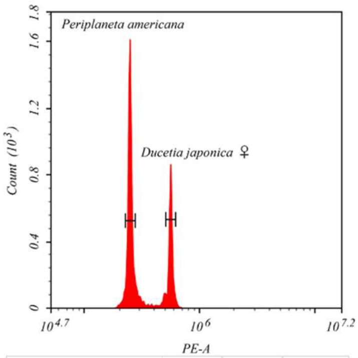

Species	Mean	CV	C-value
Periplaneta americana	252,787	3.66%	3.41
Ducetia japonica	568,836	3.56%	7.6734

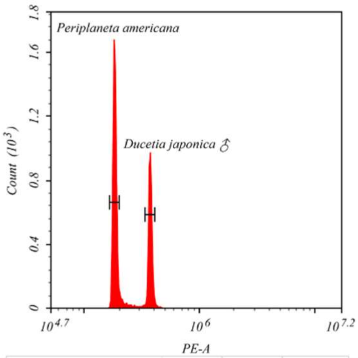

Species	Mean	CV	C-value
Periplaneta americana	186,195	3.09%	3.41
Ducetia japonica	379,192	3.06%	6.9446

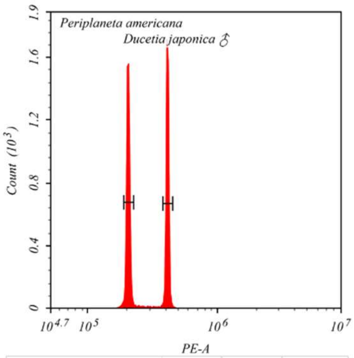

Species	Mean	CV	C-value
Periplaneta americana	207,295	2.55%	3.41
Ducetia japonica	417,630	2.19%	6.8700

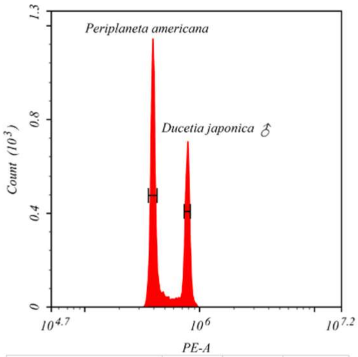

Species	Mean	CV	C-value
Periplaneta americana	397,406	3.44%	3.41
Ducetia japonica	792,112	3.33%	6.7968

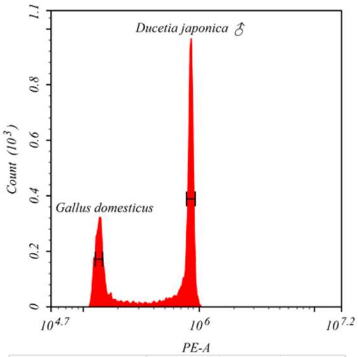

Species	Mean	CV	C-value
Gallus domesticus	137,731	4.13%	1.165
Ducetia japonica	860,483	3.82%	7.2784

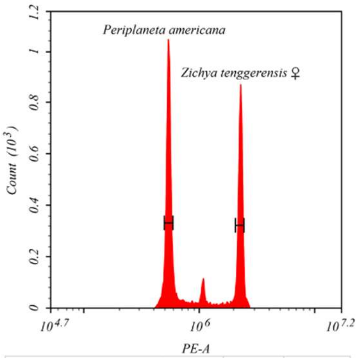

Species	Mean	CV	C-value
Periplaneta americana	549,504	3.85%	3.41
Zichya tenggerensis	2,301,488	3.47%	14.2821

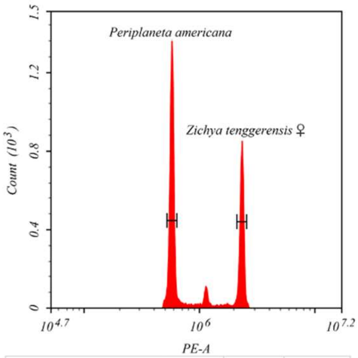

Species	Mean	CV	C-value
Periplaneta americana	582,207	3.45%	3.41
Zichya tenggerensis	2,357,293	3.42%	13.8067

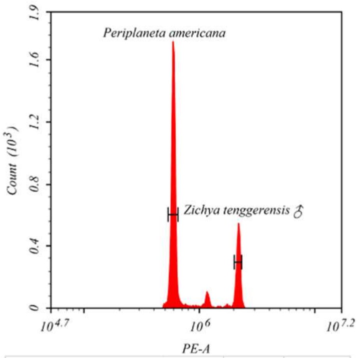

Species	Mean	CV	C-value
Periplaneta americana	600,722	3.38%	3.41
Zichya tenggerensis	2,203,489	3.25%	12.5081

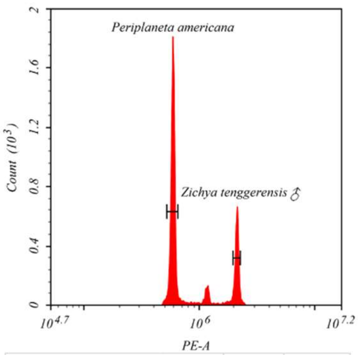

Species	Mean	CV	C-value
Periplaneta americana	596,457	3.53%	3.41
Zichya tenggerensis	2,144,952	3.13%	12.2629

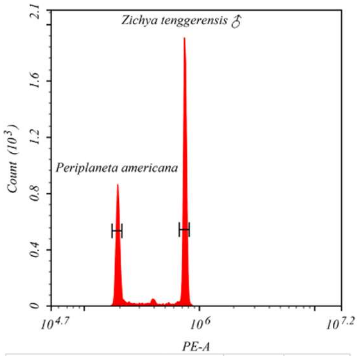

Species	Mean	CV	C-value
Periplaneta americana	197,613	3.28%	3.41
Zichya tenggerensis	757,315	2.74%	13.0682

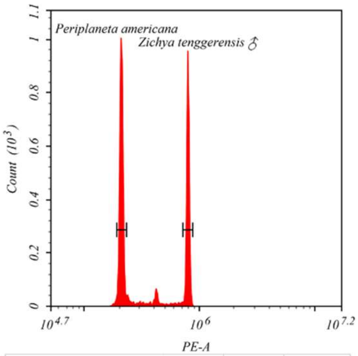

Species	Mean	CV	C-value
Periplaneta americana	212,740	3.04%	3.41
Zichya tenggerensis	805,584	2.47%	12.9127

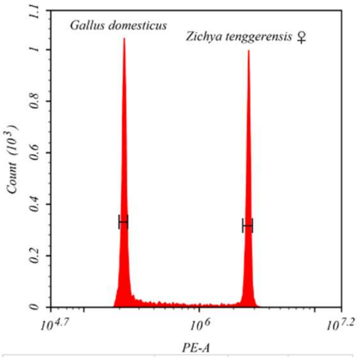

Species	Mean	CV	C-value
Gallus domesticus	224,383	3.79%	1.165
Zichya tenggerensis	2,695,455	3.57%	13.9948

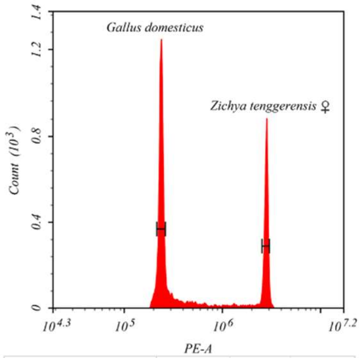

Species	Mean	CV	C-value
Gallus domesticus	240,934	3.89%	1.165
Zichya tenggerensis	2,837,998	3.52%	13.7227

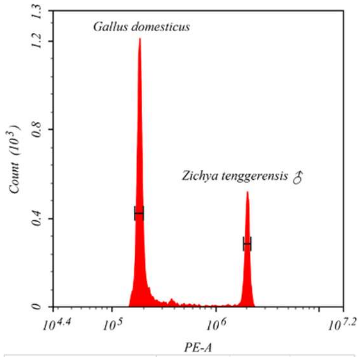

Species	Mean	CV	C-value
Gallus domesticus	186,776	4.29%	1.165
Zichya tenggerensis	2,048,275	3.67%	12.7759

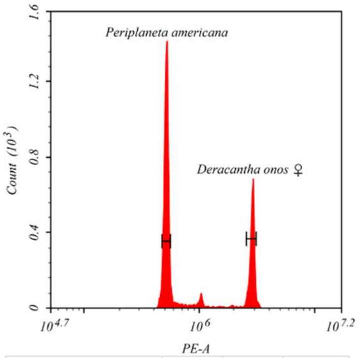

Species	Mean	CV	C-value
Periplaneta americana	523,323	3.67%	3.41
Deracantha onos	2,915,748	3.79%	18.9992

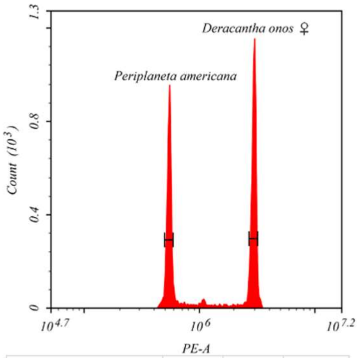

Species	Mean	CV	C-value
Periplaneta americana	551,330	3.66%	3.41
Deracantha onos	2,997,625	3.78%	18.5404

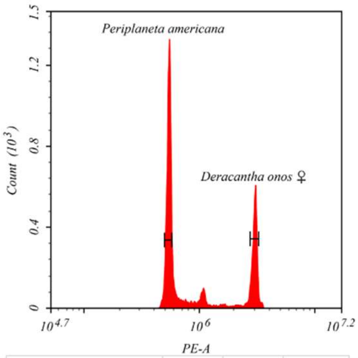

Species	Mean	CV	C-value
Periplaneta americana	549,148	3.43%	3.41
Deracantha onos	3,059,176	3.89%	18.9963

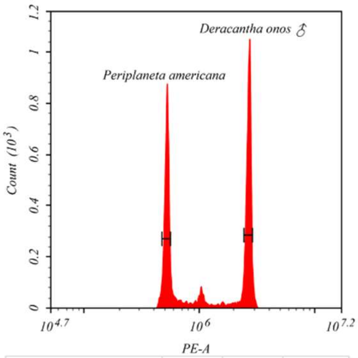

Species	Mean	CV	C-value
Periplaneta americana	528,075	3.76%	3.41
Deracantha onos	2,718,148	3.84%	17.5522

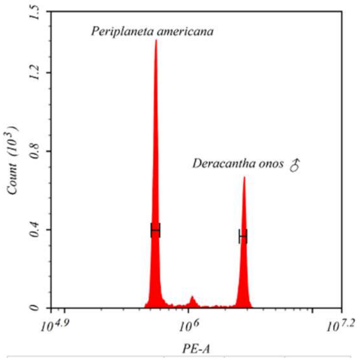

Species	Mean	CV	C-value
Periplaneta americana	549,557	3.40%	3.41
Deracantha onos	2,813,009	3.46%	17.4547

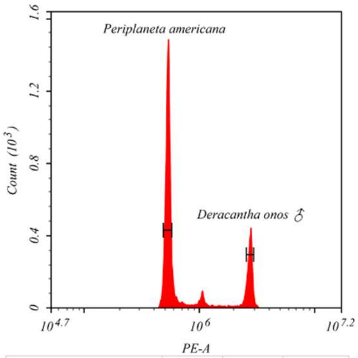

Species	Mean	CV	C-value
Periplaneta americana	537,910	3.56%	3.41
Deracantha onos	2,791,592	3.59%	17.6969

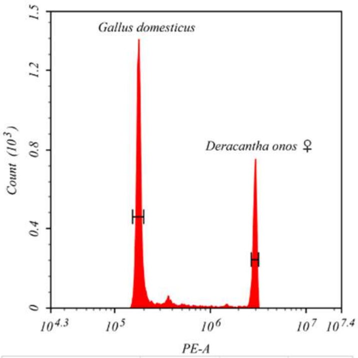

Species	Mean	CV	C-value
Gallus domesticus	181,067	4.54%	1.165
Deracantha onos	2,981,586	3.55%	19.1838

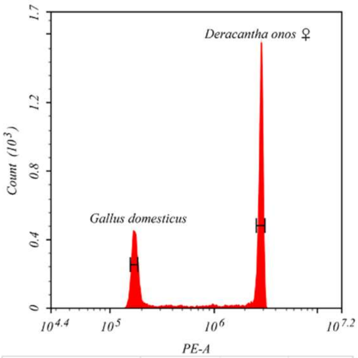

Species	Mean	CV	C-value
Gallus domesticus	173,414	4.33%	1.165
Deracantha onos	2,901,393	3.79%	19.4916

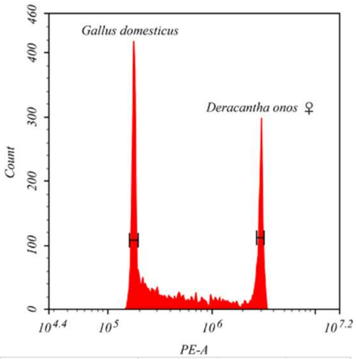

Species	Mean	CV	C-value
Gallus domesticus	179,958	3.69%	1.165
Deracantha onos	3,027,450	3.64%	19.5989

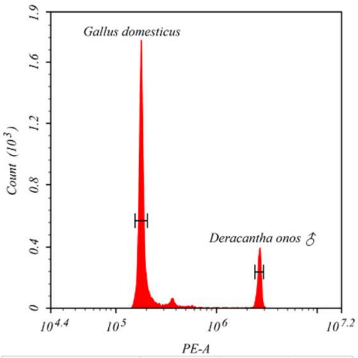

Species	Mean	CV	C-value
Gallus domesticus	182,400	4.79%	1.165
Deracantha onos	2,689,659	3.58%	17.1790

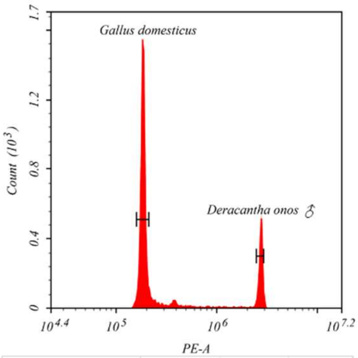

Species	Mean	CV	C-value
Gallus domesticus	188,594	4.78%	1.165
Deracantha onos	2,765,589	3.58%	17.0838

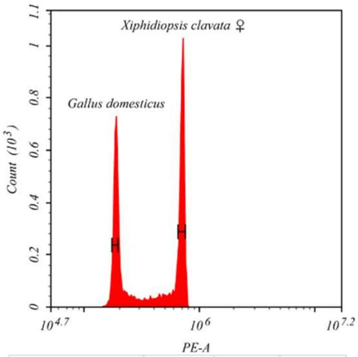

Species	Mean	CV	C-value
Gallus domesticus	189,049	3.23%	1.165
Xiphidiopsis clavata	715,884	3.56%	4.4116

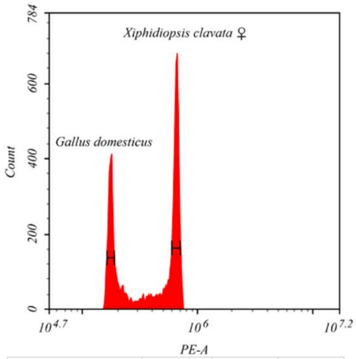

Species	Mean	CV	C-value
Gallus domesticus	179,652	3.48%	1.165
Xiphidiopsis clavata	669,639	4.05%	4.3424

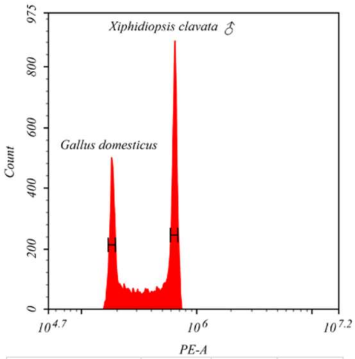

Species	Mean	CV	C-value
Gallus domesticus	185,941	3.43%	1.165
Xiphidiopsis clavata	649,140	3.77%	4.0671

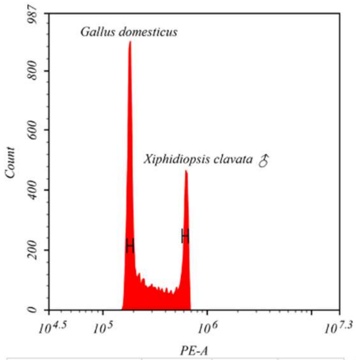

Species	Mean	CV	C-value
Gallus domesticus	185,384	3.36%	1.165
Xiphidiopsis clavata	638,475	3.22%	4.0123

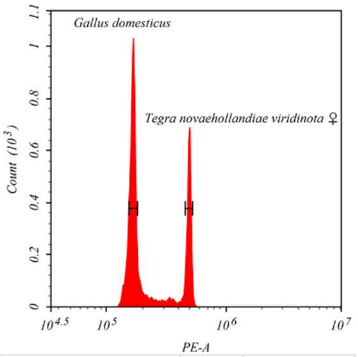

Species	Mean	CV	C-value
Gallus domesticus	168,998	3.78%	1.165
Tegra novaehollandiae viridinotata	498,219	3.14%	3.4345

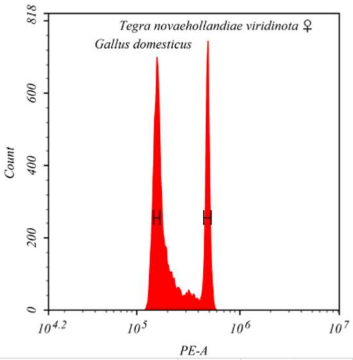

Species	Mean	CV	C-value
Gallus domesticus	158,900	4.17%	1.165
Tegra novaehollandiae viridinotata	493,753	3.25%	3.6200

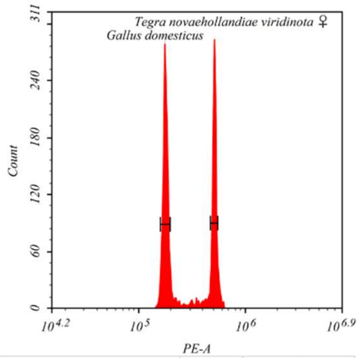

Species	Mean	CV	C-value
Gallus domesticus	179,214	4.28%	1.165
Tegra novaehollandiae viridinotata	517,390	3.09%	3.3633

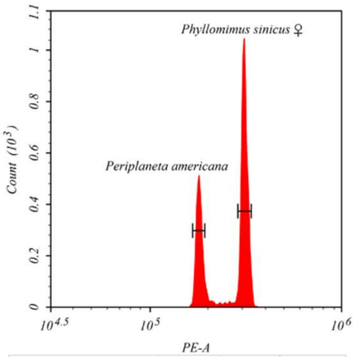

Species	Mean	CV	C-value
Periplaneta americana	182,007	2.97%	3.41
Phyllomimus sinicus	313,449	3.33%	5.8726

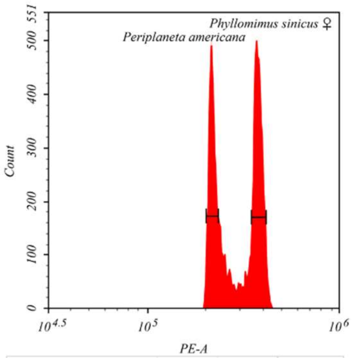

Species	Mean	CV	C-value
Periplaneta americana	218,743	3.42%	3.41
Phyllomimus sinicus	379,314	4.15%	5.9132

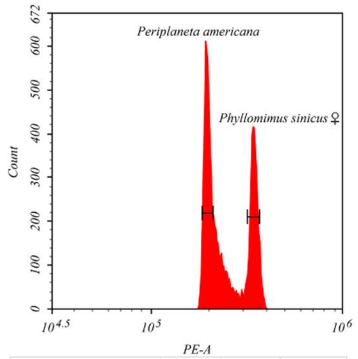

Species	Mean	CV	C-value
Periplaneta americana	198,154	3.20%	3.41
Phyllomimus sinicus	345,898	3.45%	5.9525

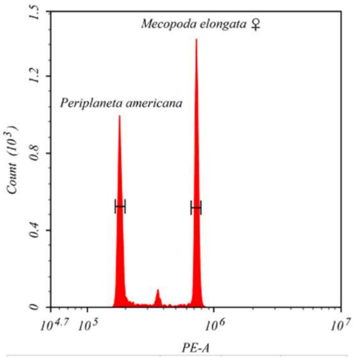

Species	Mean	CV	C-value
Periplaneta americana	182,765	3.34%	3.41
Mecopoda elongata	735,265	2.89%	13.7185

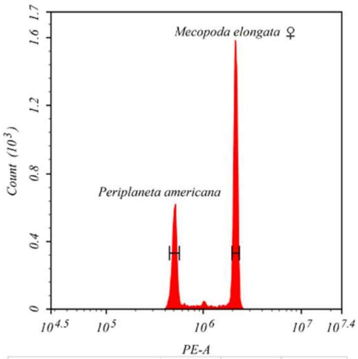

Species	Mean	CV	C-value
Periplaneta americana	513,345	4.44%	3.41
Mecopoda elongata	2,163,626	3.69%	14.3723

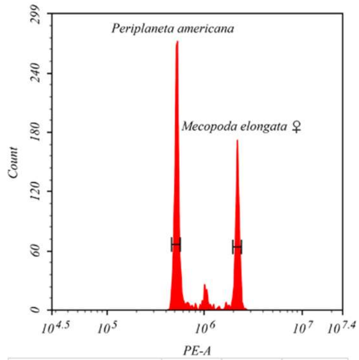

Species	Mean	CV	C-value
Periplaneta americana	526,766	4.02%	3.41
Mecopoda elongata	2,213,486	4.08%	14.3289

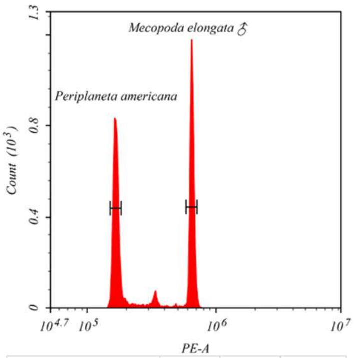

Species	Mean	CV	C-value
Periplaneta americana	167,699	3.93%	3.41
Mecopoda elongata	656,800	3.15%	13.3554

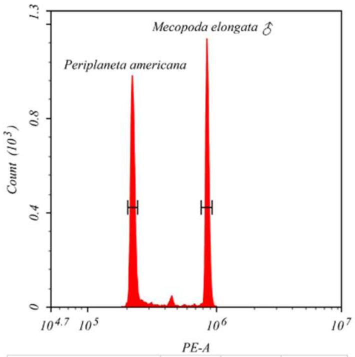

Species	Mean	CV	C-value
Periplaneta americana	226,476	3.16%	3.41
Mecopoda elongata	865,880	2.76%	13.0374

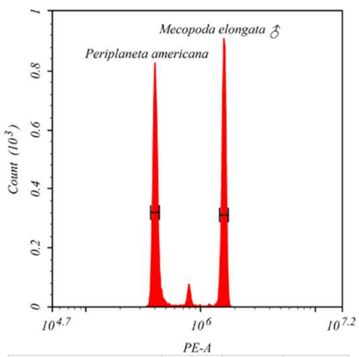

Species	Mean	CV	C-value
Periplaneta americana	404,985	3.79%	3.41
Mecopoda elongata	1,606,783	3.28%	13.5292

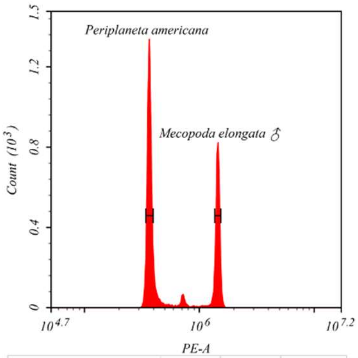

Species	Mean	CV	C-value
Periplaneta americana	368,964	3.33%	3.41
Mecopoda elongata	1,453,624	2.98%	13.4345

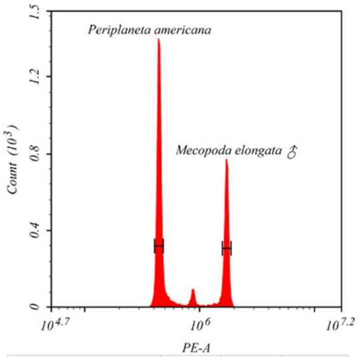

Species	Mean	CV	C-value
Periplaneta americana	446,404	3.35%	3.41
Mecopoda elongata	1,724,123	3.08%	13.1703

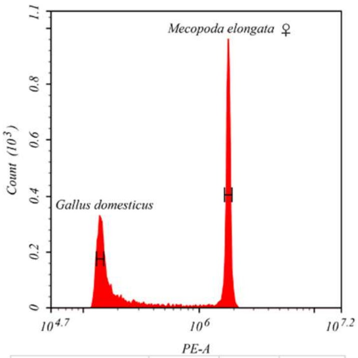

Species	Mean	CV	C-value
Gallus domesticus	141,162	4.15%	1.165
Mecopoda elongata	1,817,942	3.05%	15.0033

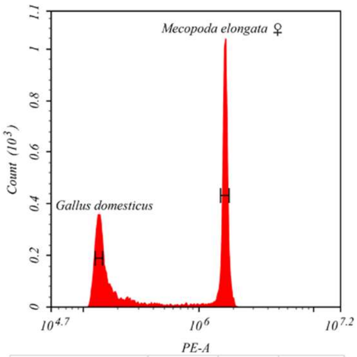

Species	Mean	CV	C-value
Gallus domesticus	137,661	4.23%	1.165
Mecopoda elongata	1,722,603	3.42%	14.5781

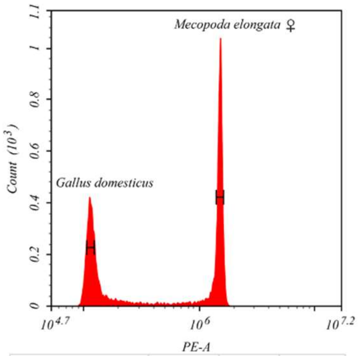

Species	Mean	CV	C-value
Gallus domesticus	115,759	4.15%	1.165
Mecopoda elongata	1,538,615	3.59%	15.4846

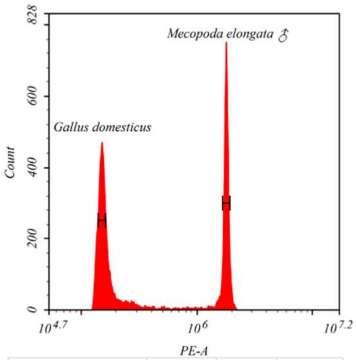

Species	Mean	CV	C-value
Gallus domesticus	151,857	4.13%	1.165
Mecopoda elongata	1,822,593	3.42%	13.9824

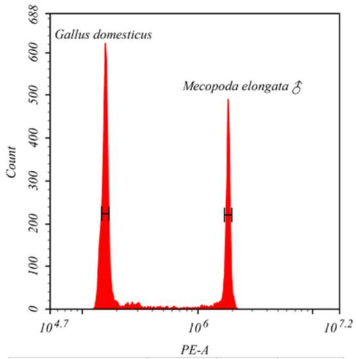

Species	Mean	CV	C-value
Gallus domesticus	159,055	3.79%	1.165
Mecopoda elongata	1,865,525	3.26%	13.6641

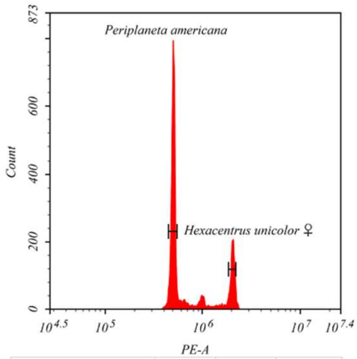

Species	Mean	CV	C-value
Periplaneta americana	509,095	3.65%	3.41
Hexacentrus unicolor	2,069,835	3.82%	13.8641

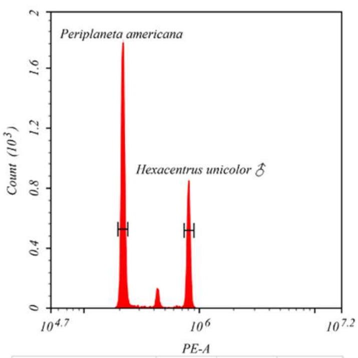

Species	Mean	CV	C-value
Periplaneta americana	219,787	3.14%	3.41
Hexacentrus unicolor	816,362	2.94%	12.6659

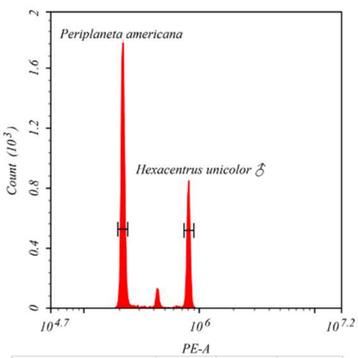

Species	Mean	CV	C-value
Periplaneta americana	207,009	3.25%	3.41
Hexacentrus unicolor	766,770	3.09%	12.6308

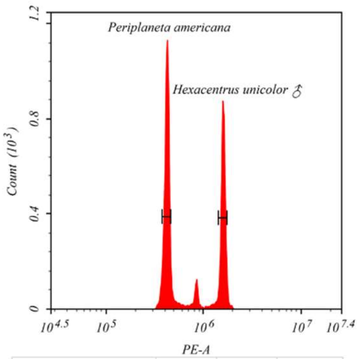

Species	Mean	CV	C-value
Periplaneta americana	430,309	4.45%	3.41
Hexacentrus unicolor	1,620,372	3.83%	12.8407

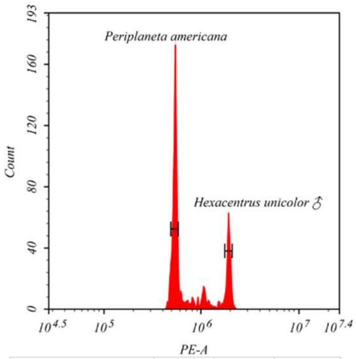

Species	Mean	CV	C-value
Periplaneta americana	546,498	4.11%	3.41
Hexacentrus unicolor	1,944,805	3.78%	12.1351

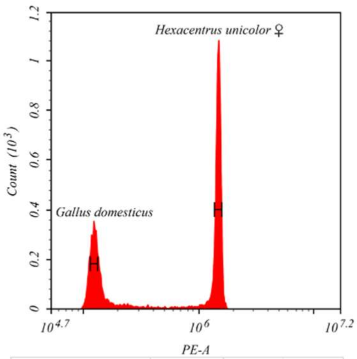

Species	Mean	CV	C-value
Gallus domesticus	123,946	4.21%	1.165
Hexacentrus unicolor	1,505,669	3.73%	14.1522

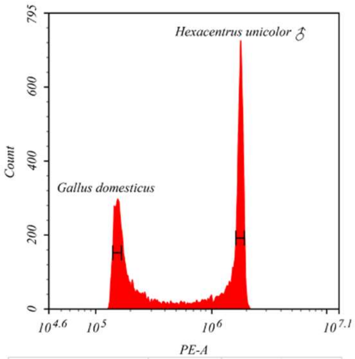

Species	Mean	CV	C-value
Gallus domesticus	154,336	4.22%	1.165
Hexacentrus unicolor	1,810,383	4.14%	13.6656

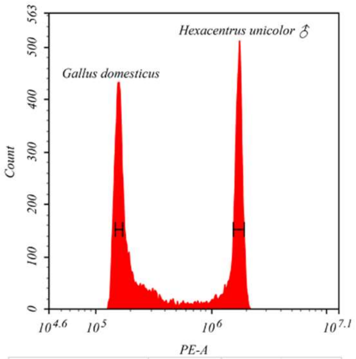

Species	Mean	CV	C-value
Gallus domesticus	158,966	4.17%	1.165
Hexacentrus unicolor	1,756,222	4.98%	12.8707

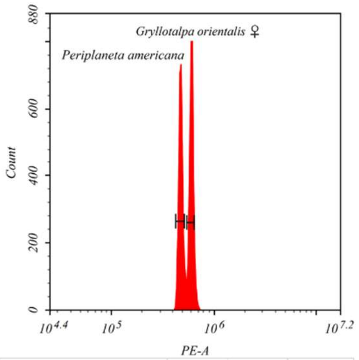

Species	Mean	CV	C-value
Periplaneta americana	482,784	3.76%	3.41
Gryllotalpa orientalis Burmeister	614,139	3.48%	4.3378

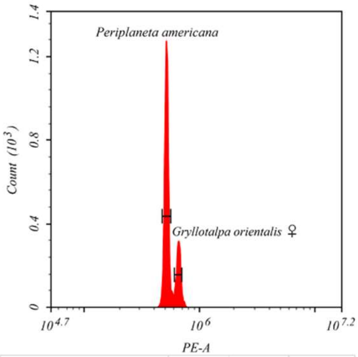

Species	Mean	CV	C-value
Periplaneta americana	522,649	3.35%	3.41
Gryllotalpa orientalis Burmeister	663,170	3.31%	4.3268

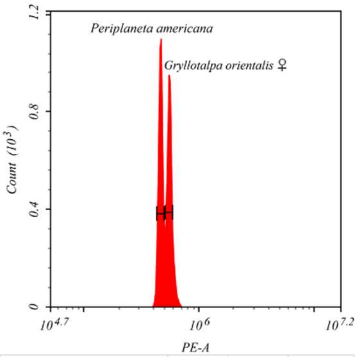

Species	Mean	CV	C-value
Periplaneta americana	468,754	3.32%	3.41
Gryllotalpa orientalis Burmeister	557,417	3.40%	4.0550

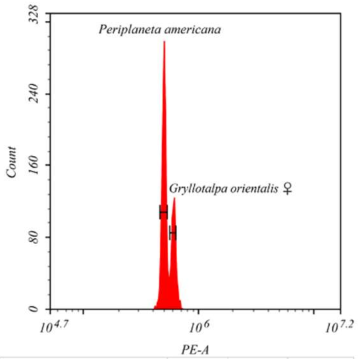

Species	Mean	CV	C-value
Periplaneta americana	505,923	3.32%	3.41
Gryllotalpa orientalis Burmeister	611,543	3.35%	4.1219

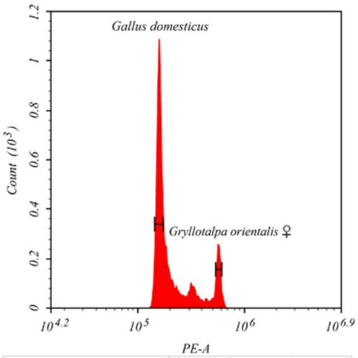

Species	Mean	CV	C-value
Gallus domesticus	159,304	4.47%	1.165
Gryllotalpa orientalis Burmeister	577,933	3.39%	4.2265

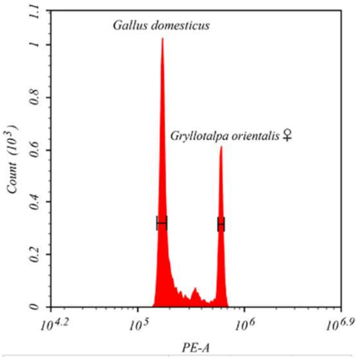

Species	Mean	CV	C-value
Gallus domesticus	170,938	4.38%	1.165
Gryllotalpa orientalis Burmeister	610,305	3.22%	4.1594

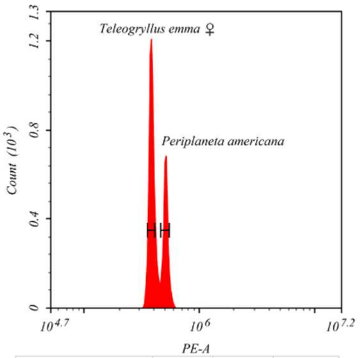

Species	Mean	CV	C-value
Periplaneta americana	514,485	3.79%	3.41
Teleogryllus emma	387,572	3.69%	2.5688

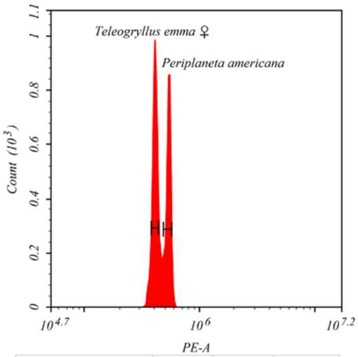

Species	Mean	CV	C-value
Periplaneta americana	545,507	3.86%	3.41
Teleogryllus emma	416,394	3.89%	2.6029

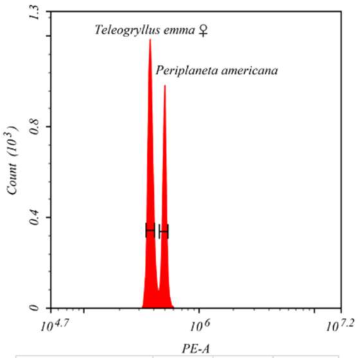

Species	Mean	CV	C-value
Periplaneta americana	505,097	3.39%	3.41
Teleogryllus emma	380,271	3.76%	2.5673

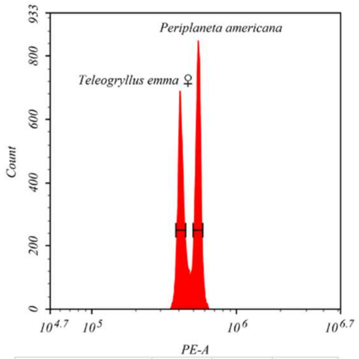

Species	Mean	CV	C-value
Periplaneta americana	546,332	3.57%	3.41
Teleogryllus emma	414,807	3.74%	2.5891

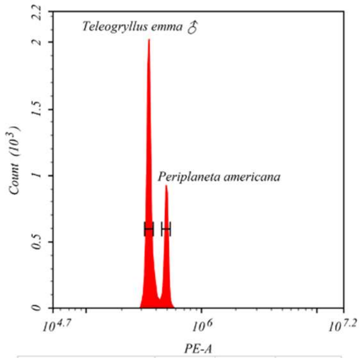

Species	Mean	CV	C-value
Periplaneta americana	500,839	3.29%	3.41
Teleogryllus emma	352,346	3.55%	2.3990

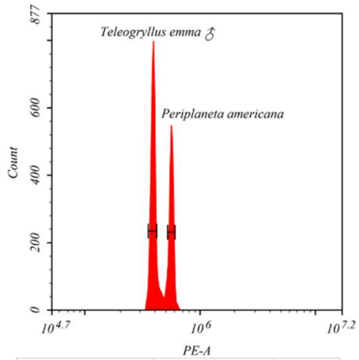

Species	Mean	CV	C-value
Periplaneta americana	568,981	3.49%	3.41
Teleogryllus emma	394,288	3.61%	2.3630

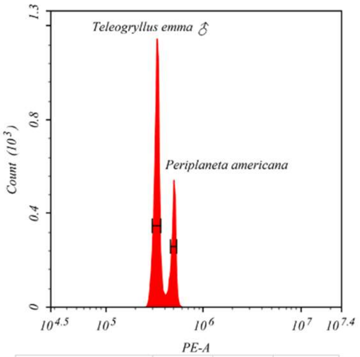

Species	Mean	CV	C-value
Periplaneta americana	507,733	3.71%	3.41
Teleogryllus emma	339,887	4.55%	2.2827

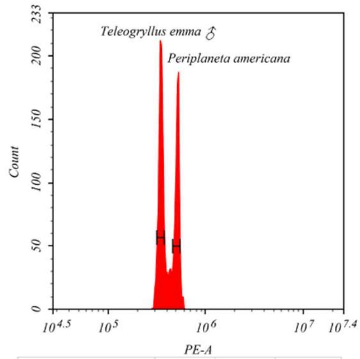

Species	Mean	CV	C-value
Periplaneta americana	521,699	3.73%	3.41
Teleogryllus emma	355,039	4.26%	2.3207

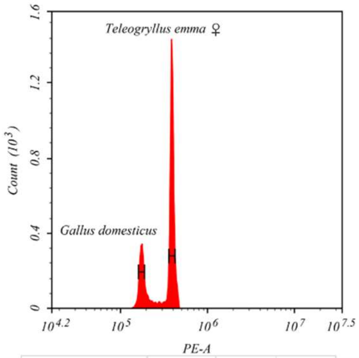

Species	Mean	CV	C-value
Gallus domesticus	177,768	4.32%	1.165
Teleogryllus emma	398,835	3.72%	2.6138

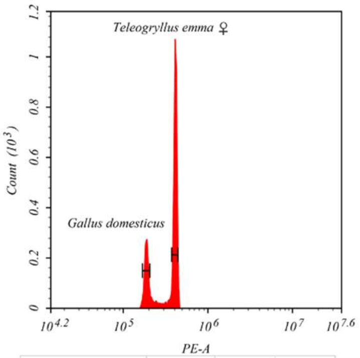

Species	Mean	CV	C-value
Gallus domesticus	191,244	4.08%	1.165
Teleogryllus emma	419,236	3.53%	2.5539

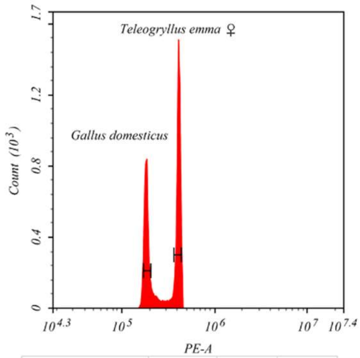

Species	Mean	CV	C-value
Gallus domesticus	188,063	4.10%	1.165
Teleogryllus emma	419,083	3.73%	2.5961

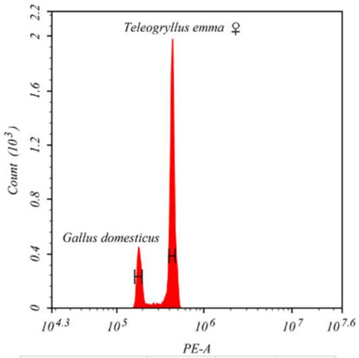

Species	Mean	CV	C-value
Gallus domesticus	183,364	4.30%	1.165
Teleogryllus emma	437,526	4.09%	2.7798

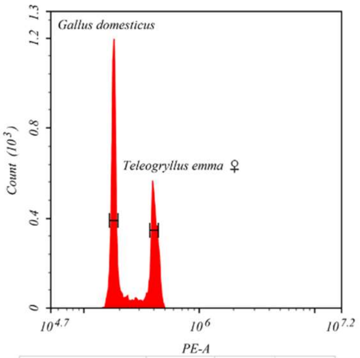

Species	Mean	CV	C-value
Gallus domesticus	182,258	3.51%	1.165
Teleogryllus emma	411,977	4.43%	2.6334

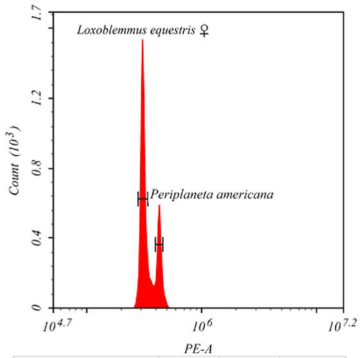

Species	Mean	CV	C-value
Periplaneta americana	432,466	3.57%	3.41
Loxoblemmus equestris	312,626	3.88%	2.4651

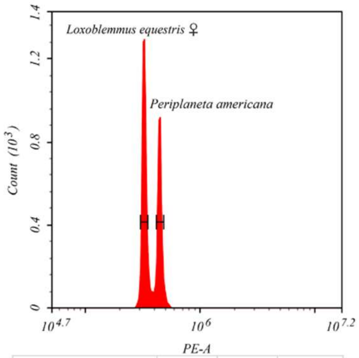

Species	Mean	CV	C-value
Periplaneta americana	449,918	3.18%	3.41
Loxoblemmus equestris	330,063	3.38%	2.5016

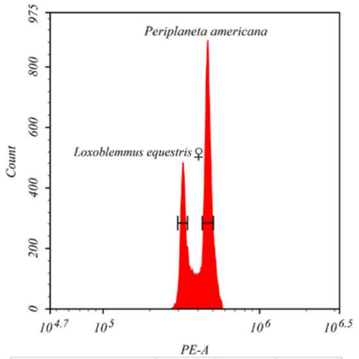

Species	Mean	CV	C-value
Periplaneta americana	470,215	3.81%	3.41
Loxoblemmus equestris	327,559	3.42%	2.3755

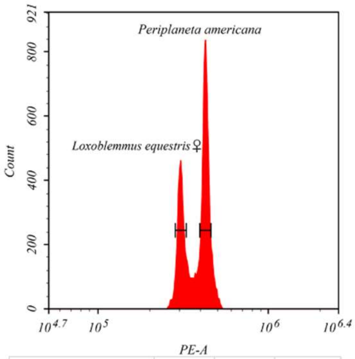

Species	Mean	CV	C-value
Periplaneta americana	430,347	3.49%	3.41
Loxoblemmus equestris	308,206	3.49%	2.4422

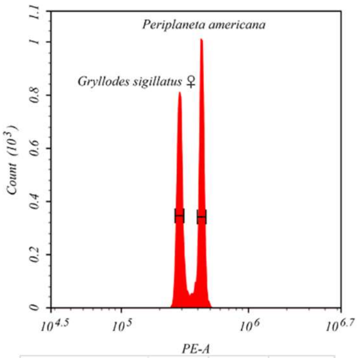

Species	Mean	CV	C-value
Periplaneta americana	432,501	3.38%	3.41
Gryllodes sigillatus	289,908	3.69%	2.2857

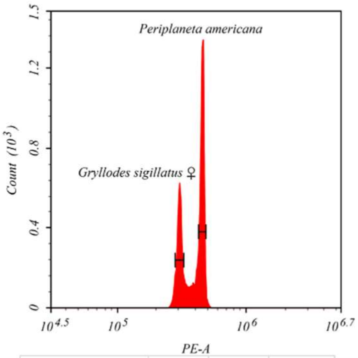

Species	Mean	CV	C-value
Periplaneta americana	461,968	2.83%	3.41
Gryllodes sigillatus	307,018	3.66%	2.2662

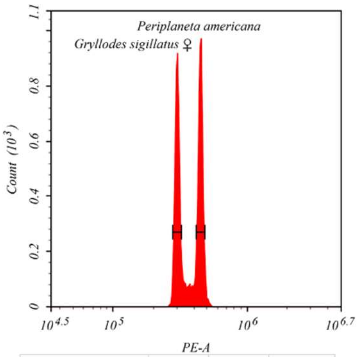

Species	Mean	CV	C-value
Periplaneta americana	454,318	3.45%	3.41
Gryllodes sigillatus	304,268	3.37%	2.2838

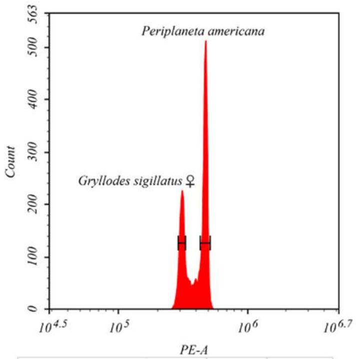

Species	Mean	CV	C-value
Periplaneta americana	476,811	3.32%	3.41
Gryllodes sigillatus	314,340	3.41%	2.2481

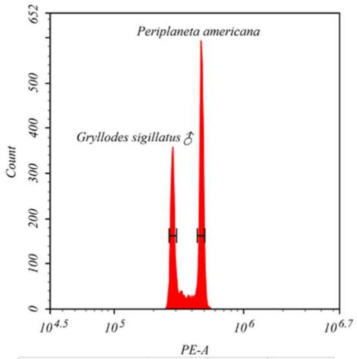

Species	Mean	CV	C-value
Periplaneta americana	479,118	2.88%	3.41
Gryllodes sigillatus	285,767	2.67%	2.0339

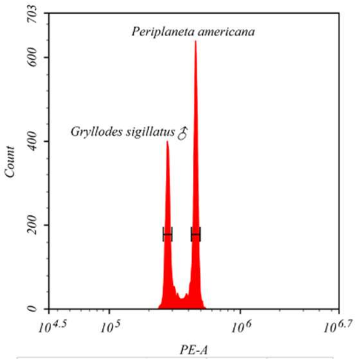

Species	Mean	CV	C-value
Periplaneta americana	458,673	2.99%	3.41
Gryllodes sigillatus	280,004	2.98%	2.0817

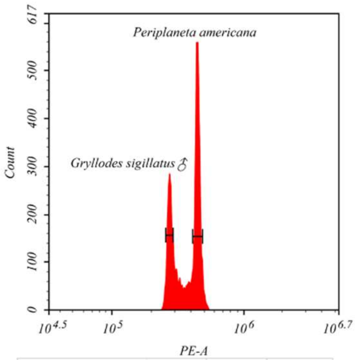

Species	Mean	CV	C-value
Periplaneta americana	453,464	3.60%	3.41
Gryllodes sigillatus	278,004	3.16%	2.0906

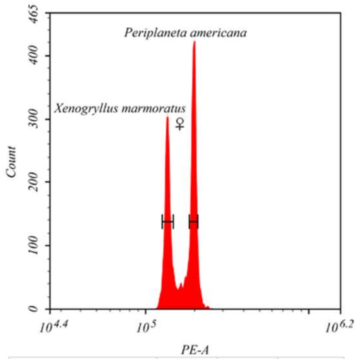

Species	Mean	CV	C-value
Periplaneta americana	199,108	2.56%	3.41
Xenogryllus marmoratus	137,633	3.36%	2.3572

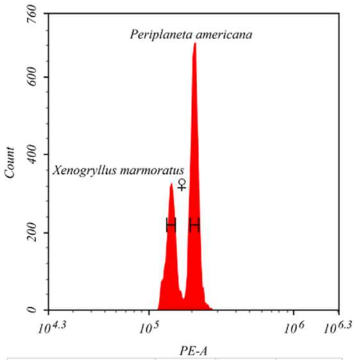

Species	Mean	CV	C-value
Periplaneta americana	209,120	3.56%	3.41
Xenogryllus marmoratus	143,773	4.45%	2.3444

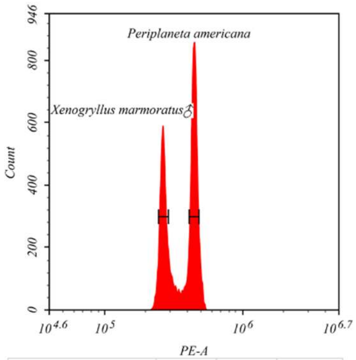

Species	Mean	CV	C-value
Periplaneta americana	450,565	3.88%	3.41
Xenogryllus marmoratus	267,436	3.95%	2.0240

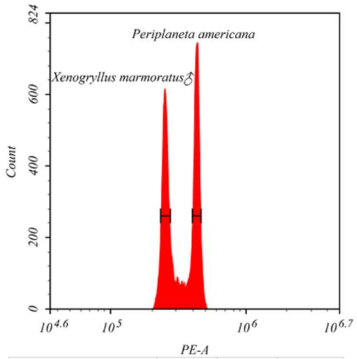

Species	Mean	CV	C-value
Periplaneta americana	431,416	3.60%	3.41
Xenogryllus marmoratus	252,444	3.98%	1.9954

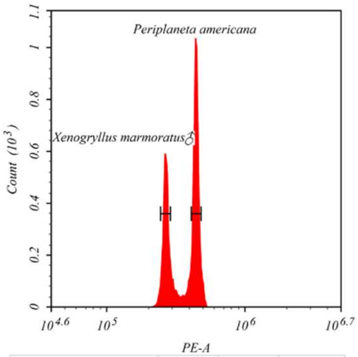

Species	Mean	CV	C-value
Periplaneta americana	445,632	3.56%	3.41
Xenogryllus marmoratus	268,738	3.69%	2.0564

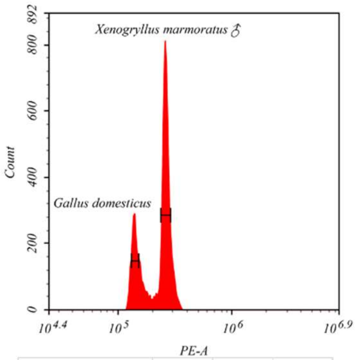

Species	Mean	CV	C-value
Gallus domesticus	142,814	4.14%	1.165
Xenogryllus marmoratus	264,449	4.56%	2.1572

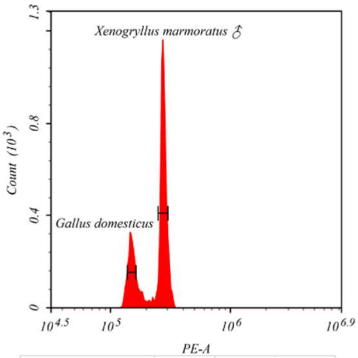

Species	Mean	CV	C-value
Gallus domesticus	151,255	4.07%	1.165
Xenogryllus marmoratus	279,773	4.21%	2.1549

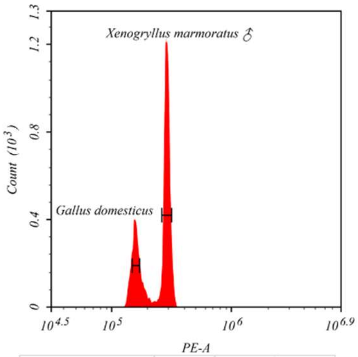

Species	Mean	CV	C-value
Gallus domesticus	159,806	4.01%	1.165
Xenogryllus marmoratus	292,880	3.97%	2.1351

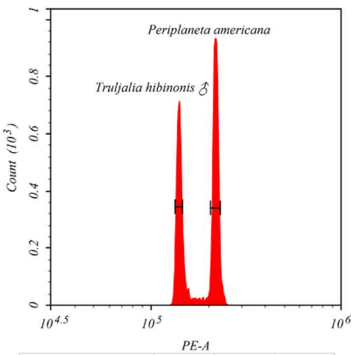

Species	Mean	CV	C-value
Periplaneta americana	220,375	2.61%	3.41
Truljalia hibinonis	140,738	2.13%	2.1777

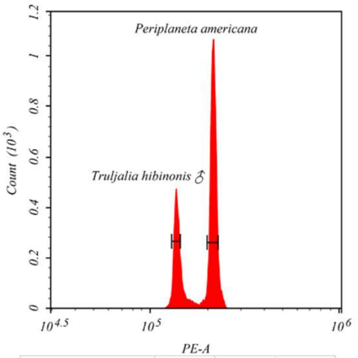

Species	Mean	CV	C-value
Periplaneta americana	216,831	2.75%	3.41
Truljalia hibinonis	137,979	2.28%	2.1699

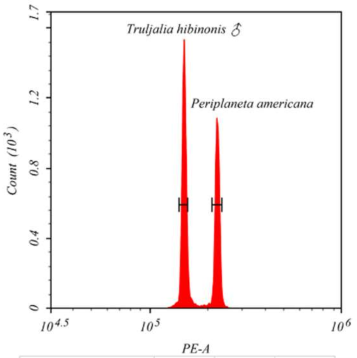

Species	Mean	CV	C-value
Periplaneta americana	226,341	2.17%	3.41
Truljalia hibinonis	152,353	2.09%	2.2953

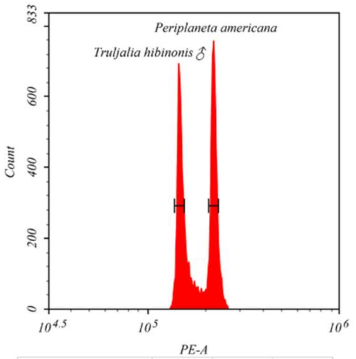

Species	Mean	CV	C-value
Periplaneta americana	222,244	2.69%	3.41
Truljalia hibinonis	148,260	2.43%	2.2748

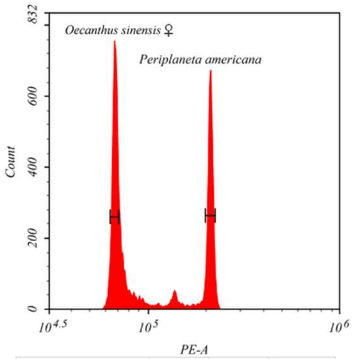

Species	Mean	CV	C-value
Periplaneta americana	212,909	2.41%	3.41
Oecanthus sinensis	68,089	2.44%	1.0905

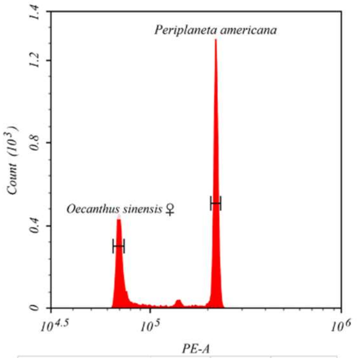

Species	Mean	CV	C-value
Periplaneta americana	222,798	2.13%	3.41
Oecanthus sinensis	70,039	2.68%	1.0720

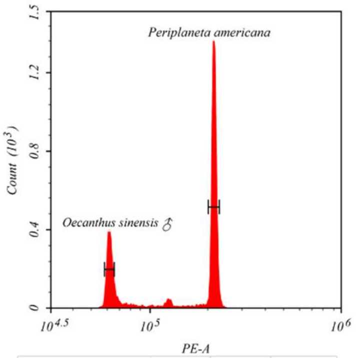

Species	Mean	CV	C-value
Periplaneta americana	218,080	2.25%	3.41
Oecanthus sinensis	62,594	2.66%	0.9787

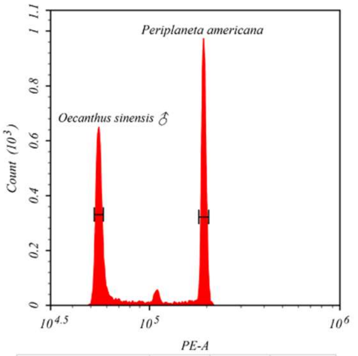

Species	Mean	CV	C-value
Periplaneta americana	195,039	2.38%	3.41
Oecanthus sinensis	55,411	2.90%	0.9688

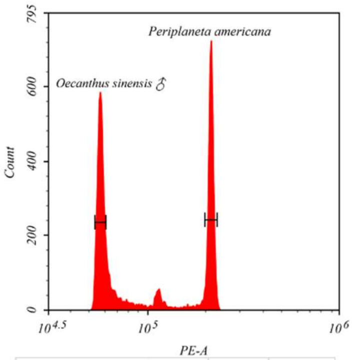

Species	Mean	CV	C-value
Periplaneta americana	215,673	2.72%	3.41
Oecanthus sinensis	57,507	2.94%	0.9092

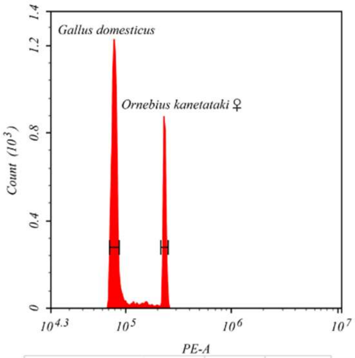

Species	Mean	CV	C-value
Gallus domesticus	79,712	4.58%	1.165
Ornebius kanetataki	237,647	2.59%	3.4732

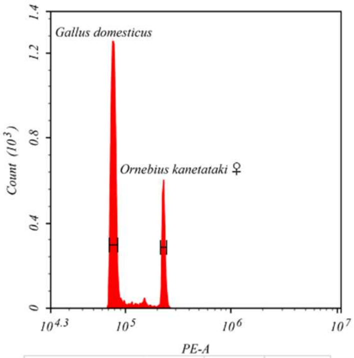

Species	Mean	CV	C-value
Gallus domesticus	78,485	4.13%	1.165
Ornebius kanetataki	233,939	2.75%	3.4725

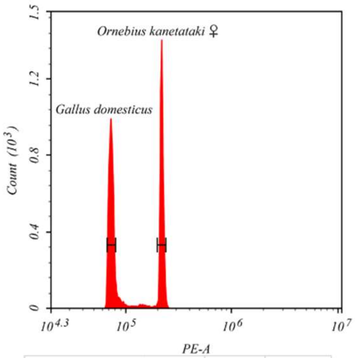

Species	Mean	CV	C-value
Gallus domesticus	74,058	4.11%	1.165
Ornebius kanetataki	222,920	2.93%	3.5067

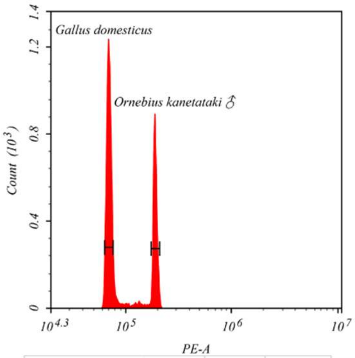

Species	Mean	CV	C-value
Gallus domesticus	71,027	4.33%	1.165
Ornebius kanetataki	193,534	3.43%	3.1744

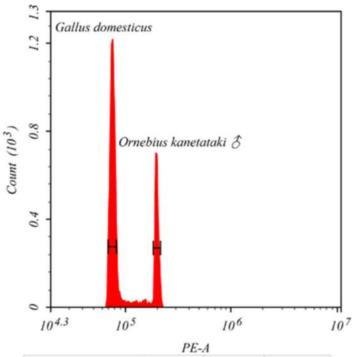

Species	Mean	CV	C-value
Gallus domesticus	77,076	4.51%	1.165
Ornebius kanetataki	201,494	3.09%	3.0456

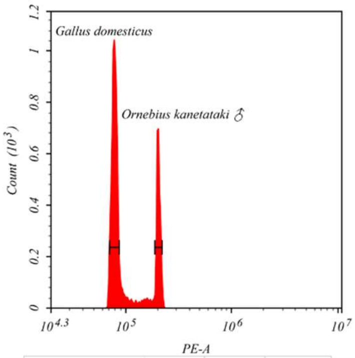

Species	Mean	CV	C-value
Gallus domesticus	79,878	4.76%	1.165
Ornebius kanetataki	207,546	3.34%	3.0270

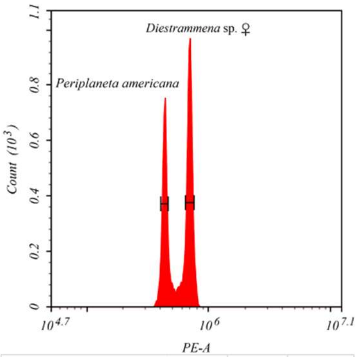

Species	Mean	CV	C-value
Periplaneta americana	440,314	3.43%	3.41
Diestrammena sp.	706,779	3.53%	5.4736

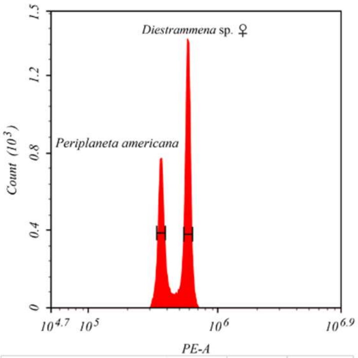

Species	Mean	CV	C-value
Periplaneta americana	367,710	3.45%	3.41
Diestrammena sp.	590,874	3.41%	5.4795

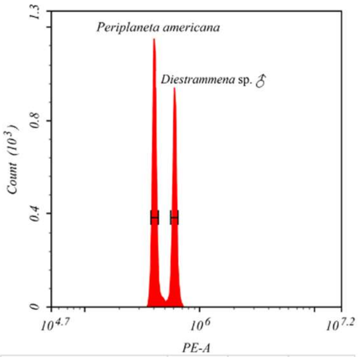

Species	Mean	CV	C-value
Periplaneta americana	408,934	3.18%	3.41
Diestrammena sp.	613,070	3.03%	5.1122

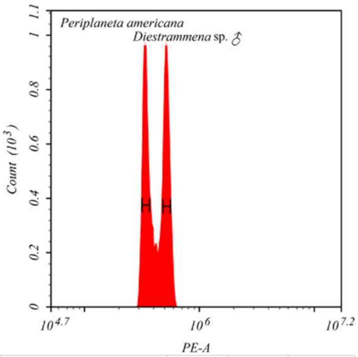

Species	Mean	CV	C-value
Periplaneta americana	344,227	3.50%	3.41
Diestrammena sp.	522,670	3.94%	5.1777

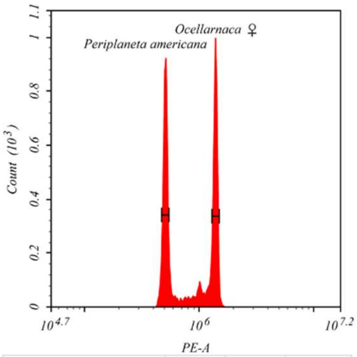

Species	Mean	CV	C-value
Periplaneta americana	511,473	3.46%	3.41
Ocellarnaca sp.	1,395,840	3.20%	9.3061

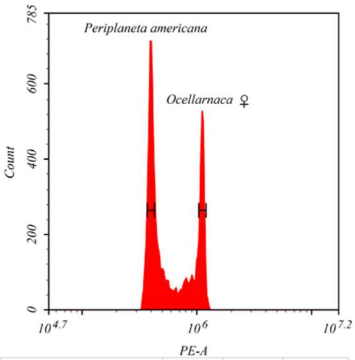

Species	Mean	CV	C-value
Periplaneta americana	400,828	3.92%	3.41
Ocellarnaca sp.	1,117,004	3.36%	9.5028

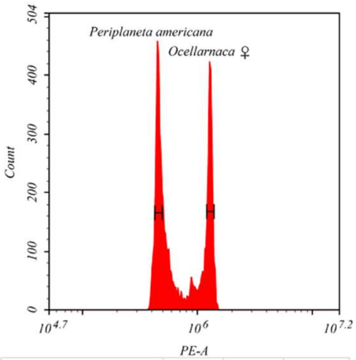

Species	Mean	CV	C-value
Periplaneta americana	459,994	3.81%	3.41
Ocellarnaca sp.	1,295,042	3.40%	9.6003

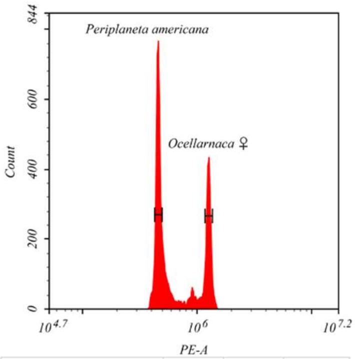

Species	Mean	CV	C-value
Periplaneta americana	460,430	3.51%	3.41
Ocellarnaca sp.	1,265,767	3.39%	9.3744

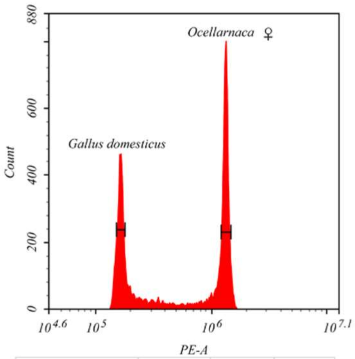

Species	Mean	CV	C-value
Gallus domesticus	165,894	3.92%	1.165
Ocellarnaca sp.	1,349,034	4.35%	9.4737
